# Circular rubber aggregate CFST stub columns under axial compression: prediction and reliability analysis

**DOI:** 10.1038/s41598-024-74990-5

**Published:** 2024-10-31

**Authors:** Khaled Megahed, Nabil Said Mahmoud, Saad Elden Mostafa Abd-Rabou

**Affiliations:** Department of Structural Engineering, Mansoura University, PO BOX 35516, Mansoura, Egypt

**Keywords:** Rubber aggregate concrete, Concrete-filled steel tubes, Stub columns, Symbolic regression, XGBoost, CatBoost, Random forest, LightGBM, Gaussian process regression, Bayesian optimization (BO) technique, Reliability analysis, Civil engineering, Computer science, Scientific data, Statistics

## Abstract

Extensive studies support using steel tubes to enhance the structural integrity of rubber aggregate concrete (RBAC), namely RBAC-filled steel tubes (RCFST). However, current design codes for assessing the axial compressive behaviour of circular stub RCFST (CS-RCFST) columns are limited. Furthermore, there is a scarcity of studies focused on ensuring the structural safety of these columns. Based on an extensive experimental database comprising 145 columns, this study explores machine learning (ML) capabilities for predicting the axial strength of CS-RCFST columns, using six typical machine-learning models, i.e., symbolic regression (SR), XGBoost, CatBoost, random forest, LightGBM, and Gaussian process regression models. The hyperparameter tuning of the introduced ML models is performed using the Bayesian Optimization technique. The comparison results show that the CatBoost model is the most reliable and accurate ML model (R^2^ = 0.999 and 0.993 for the training and testing sets, respectively). In addition, a simple and practical design expression for CS-RCFST columns has been developed with acceptable accuracy based on the SR model (an average test-to-prediction ratio of 0.99 and CoV of 0.132). Meanwhile, the axial strength predicted by ML models was compared with two prominent practice codes (i.e., AISC360 and EC4). The comparison results indicated that the ML models could introduce a highly reliable and accurate approach over current design standards for strength prediction. Furthermore, a reliability analysis is conducted on two different ML models to evaluate the reliability of utilising ML models in practical design applications. This assessment involves identifying the statistical properties associated with the compressive strength of RBAC, as well as introducing the required resistance design factors aligned with the target reliability recommended by code standards.

**Supplementary Information:**

The online version contains supplementary material available at 10.1038/s41598-024-74990-5.

## Introduction

The construction industry faces significant challenges in terms of sustainability, particularly concerning concrete production. Efforts have been made to mitigate the environmental impact of concrete by incorporating waste materials into its composition. Using waste materials, such as construction and demolition waste or recycled tyre rubber, offers several advantages, including reducing greenhouse gas emissions, conserving natural resources, and improving the mechanical properties and durability of concrete structures. Rubberised concrete, which incorporates rubber particles from used tyres as a partial replacement for traditional aggregates, has emerged as an environmentally sustainable solution for recycling waste tyre disposal and a highly promising material for various civil engineering applications^1–8^.

Although utilising recycled materials in concrete is beneficial, it also presents challenges, such as potential reductions in the strength and stiffness of structural members^2^. To address these challenges, a viable solution is integrating rubberised concrete with steel tubes, forming a composite member known as a rubberised concrete-filled steel tube (RCFST). Inserting rubber aggregate concrete (RBAC) inside the steel tube helps alleviate the declines in strength, stiffness, and durability performance^1–8^. The steel tube offers lateral confinement to the rubberised concrete core, resulting in a three-dimensional compression state that enhances the compression strength, ductility, and energy absorption capacity the CFST columns can provide through their composite action. Ductility is the ability of a material or structure to undergo significant plastic deformation before failure, which is particularly important in earthquake-resistant design. Energy absorption capacity refers to the ability of a material or structure to absorb and dissipate energy, making it more resilient under dynamic loads, such as those experienced during seismic events. Duarte et al.^2^ studied the impact of cross-section shape on the strength and ductility of circular stub RCFST (CS-RCFST) columns. Their findings revealed that columns with circular sections exhibited enhanced durability compared to those with square or rectangular sections. Furthermore, columns filled with rubber aggregate concrete offer superior ductility compared to those of normal concrete. This is a beneficial solution for seismic-prone areas where effective energy dissipation is critical. Abuzaid et al.^7^ conducted experimental tests on RCFST short columns with a 10% rubber replacement ratio and various thicknesses of steel tubes under axial compression. The findings reveal that the axial compressive capacity of CS-RCFST columns is only 1.4–6.6% lower than that of normal CFST short columns.

Experimental investigations are commonly used to investigate the behaviour of CFST columns. Recently, numerous studies have focused on the effects of different concrete materials^7^ and the behaviour of structures following fire exposure^2,9^. However, experimental studies are often limited by the range of parameters and can be costly and time-consuming. Machine learning (ML) techniques can complement experimental studies, as they have proven effective in predicting structural element behaviours. ML algorithms such as Gaussian process (GPR)^10,11^, gene expression programming (GEP)^12,13^, artificial neural network (ANN)^14^, and symbolic regression^15–17^ have been developed and successfully used by researchers in developing empirical formulas and statistical models for predicting the axial strength of CFST columns. For example, Ahmadi et al.^15^ utilised ANN to predict the ultimate strength of short CFST columns. In addition, Zarringol et al.^16^ utilised four distinct databases, totalling 3091 CFST columns, encompassing rectangular and circular columns with and without eccentricity. They developed four separate ANN models for the axial capacities of each category and incorporated strength reduction factors to enhance practical design applications. Khaled et al.^17^ developed several ML models for predicting the axial capacity of stub columns with circular, rectangular and double-skin cross-section shapes. They introduced simple designing equations using symbolic regression with an experimental database of 1316 specimens. Furthermore, Hou and Zhou^14^ optimised ML models, including the backpropagation ANN, GPR model, genetic algorithm, radial basis function neural network (RBFNN), and multiple linear regression (MLR) models, to predict the axial compressive strength of stub and long circular CFST columns. Guneyisi et al.^13^ utilised gene expression programming to generate empirical formulations for the axial strength of circular CFST stub columns.

While significant research has been devoted to predicting the strength of CFST columns with conventional concrete, the authors have noticed a scarcity of studies focusing on predicting the axial capacity of CS-RCFST columns and assessing their reliability. Furthermore, to the best of the author’s knowledge, there is no research specifically addressing the implementation of machine-learning techniques for CS-RCFST columns. Chen et al.^18^ investigated the reliability of incorporating recycled aggregate concrete in CFST columns; however, there remains a research gap concerning the reliability of column axial strength when replacing aggregates with recycled rubber materials. This research introduces prediction ML models for CS-RCFST columns and assesses the reliability of using these models in practical engineering. An extensive experimental database of 145 specimens of CS-RCFST columns is collected from diverse research papers under axial load without eccentricity. Six data-driven models are developed, including Gaussian process regression (GPR), symbolic regression (SR), XGBoost (XGB), CatBoost (CATB), Random Forest (RF), and LightGBM (LGBM) models. The axial loads reported from the experimental results are normalised to enhance the performance of the ML models. The hyperparameter tuning of the introduced ML models is performed using the Bayesian Optimization (BO) technique. In addition, a simple and practical design expression for CS-RCFST columns has been proposed based on the SR model. Furthermore, a reliability analysis is conducted on two different ML models to evaluate the reliability of utilising ML models in practical design applications, and their reliability results were compared with those of two code standards. The evaluation process involves identifying the statistical properties associated with the compressive strength of RBAC, as well as introducing the required resistance design factors corresponding to the target reliability recommended by code standards.

### Research significance

The significance of this research lies in its advancement of predictive methods for assessing the axial strength of Circular Stub-Rubber Aggregate Concrete-Filled Steel Tubes (CS-RCFST) columns, a crucial area with limited existing studies and practical guidance. The integration of rubber aggregate concrete (RBAC) with steel tubes has shown promising potential to enhance structural integrity. However, current design codes for evaluating CS-RCFST columns are outdated and insufficiently comprehensive. This study addresses a significant gap in the literature by leveraging machine learning (ML) techniques to predict the axial strength of these columns, offering a more reliable and accurate alternative to traditional methods.

The research employs an extensive experimental database of 145 CS-RCFST columns, from which six advanced ML models—symbolic regression (SR), XGBoost, CatBoost, random forest, LightGBM, and Gaussian process regression—are developed and tested. The study innovatively applies Bayesian Optimization for hyperparameter tuning. The comparison with prominent codes like AISC360 and EC4 highlights the potential of ML models to offer more accurate and reliable predictions than existing standards. Additionally, a practical design expression derived from the SR model provides a simple yet effective means to estimate axial strength, with high accuracy and minimal error, compared to traditional design codes. By performing a reliability analysis, this study evaluates the robustness of ML models in practical design applications, providing valuable insights into the statistical properties of RBAC and necessary design factors for target reliability.

## Experimental database

**Fig. 1:**
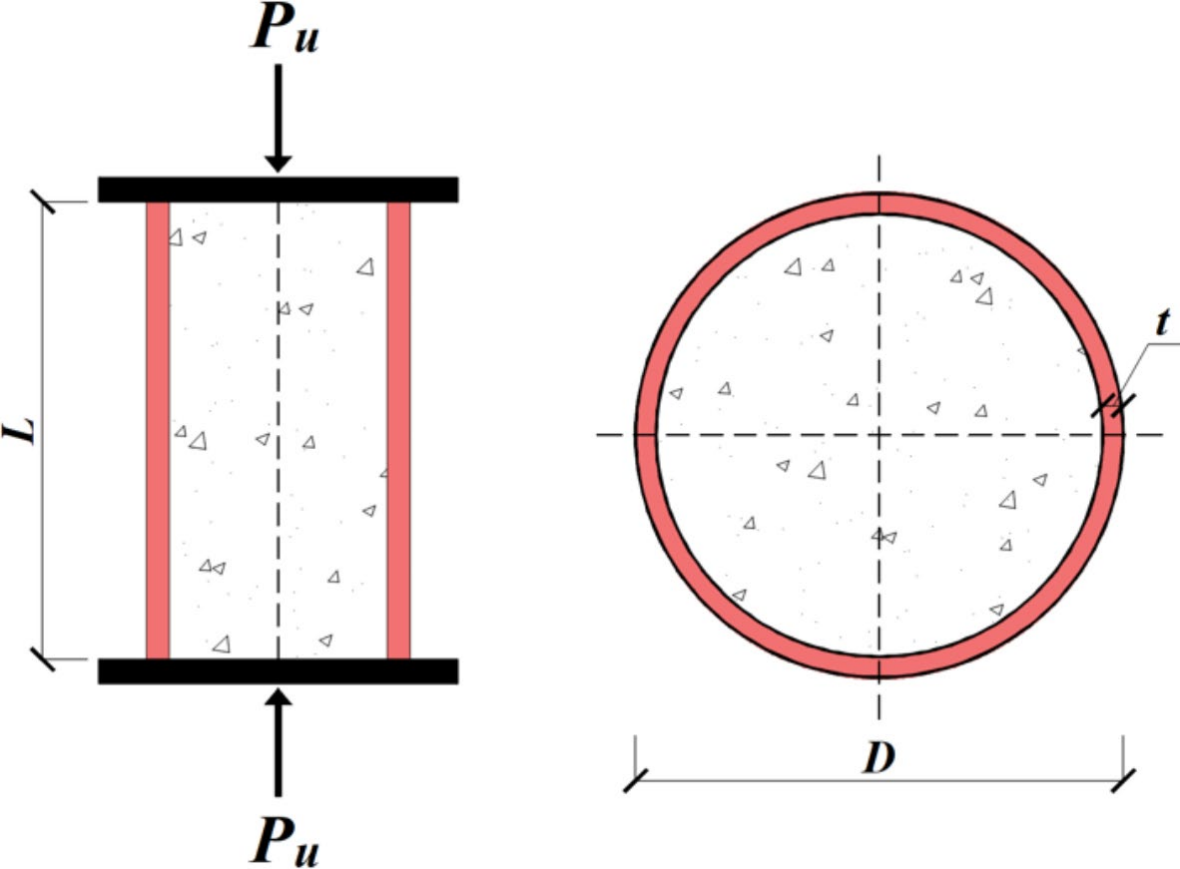
The dimensions of CS-RCFST columns.

Numerous experimental investigations have been conducted on the axial compressive behaviour of circular stub RBAC-FST (CSRCFST) columns. In this study, a comprehensive database of CSRCFST columns, including 145 experimental test results sourced from various studies^15,19–29^, is collected. Table 1 provides database outlines for each study, including six inputs, i.e., outer diameter (*D*), tube thickness (*t*), column height (*L*), steel yield strength (*f*_*y*_), cylinder compressive strength of concrete (*f*_*c*_ʹ), rubber replacement ratio (*R*), along with a single output parameter, the strength index *p*_*si*_. The schematic diagram of CS-RCFST columns is shown in Fig. 1. The strength index is extracted from normalising the axial load capacity by dividing the column axial capacity by the sum of the individual strengths of its components: the steel tubes and core concrete^17^, as follows:

1 $${p}_{si}=\frac{{P}_{u}}{{N}_{pl}}, {N}_{pl}={A}_{s}{f}_{y}+{A}_{c}{f}_{c}{\prime}$$

where *A*_*s*_ and *A*_*c*_ are the outer steel tube and concrete areas, respectively. Table 2 outlines the statistical properties of the output and input features within the established database. All the chosen specimens are stub columns with a slenderness ratio (L/D) of 4.0 or less. As shown in Table 2, the database covers a wide range of steel section slenderness. In addition, the database encompasses a wide range of concrete and steel strengths.

**Table 1:** Summary of Collected 145 CS-RCFST columns database.

Ref	*#*	*D* (*mm*)	*t* (*mm*)	*L* (*mm*)	*f*_*y*_ (*MPa*)	*f*_*c*_*′* (*MPa*)	*R*	*D/t*	*L/D*	*p*_*si*_	*P*_*u*_
^24^	16	219–219	3.0–6.0	660	215	18–36	0.1–0.3	36.5–73	3.01	1.4–1.82	1715–3310
^5^	19	86–89	2.0–3.5	188	330–342	17–86	0.0–0.1	25.4–43	2.11–2.19	0.84–1.92	409–825
^1^	3	200	2.73	600	331	47–62	0.0–0.3	73.26	3.0	1.12–1.19	2331–2748
^2^	15	114–219	2.7–4.25	300–500	284–456	20–42	0.0–0.15	35.6–57	2.28–3.29	1.05–1.27	484–2888
^25^	12	165	2.0–4.0	330	242	10–30	0.0–0.3	41.3–83	2.0	1.34–1.68	736–1435
^26^	16	114	2.0	456	382	3.6–18	0.0–0.3	57.0	4.0	1.11–1.54	340–638
^3^	12	115–165	3.0–4.0	345–495	231–298	24–39	0.0–0.3	28.8–55	3.0	1.05–1.56	759–1618
^4^	1	86.0	2.0	188	342	54.3	0.2	43.0	2.19	1.18	552
^6^	3	165.1	3.5	400	395	14–50	0.0–0.3	47.17	2.42	1.08–1.15	1130–1876
^27^	2	114.3	4.5	300–455	260	15.4	0.25	25.4	2.62–3.98	1.36–1.49	729–799
^28^	3	125	2.5	500	280	27–34	0.0–0.15	50.0	4.0	1.42–1.46	833–960
^29^	16	87.1	1.9	253–348	255	4.8–26	0.0–0.35	45.84	2.9–4.0	1.15–1.5	180–351
^7^	6	150	2.75–5.0	330	226	16–23	0.0–0.1	30–54.6	2.2	1.72–2.15	1136–1666
^30^	1	114	4.8	300	333	41.4	0.2	23.75	2.63	1.27	1146
^31^	6	152	2.8	300	290	6.4–54	0.0–0.6	54.29	1.97	1.27–1.31	618–1688
^8^	3	101.6	1.6	300	220	4.3–11.5	0.35–0.75	63.5	2.95	1.09–1.13	162–222
^32^	11	114	2.0	456	382	17–33	0.0–0.1	57.0	4.0	1.03–1.08	456–633

^#^ is the number of experimental tests for each reference.

**Table 2:** Statistic features of the experimental dataset.

Variable	Symbol	Type	Statistics					
			Min	Max	Mean	Std	Skewness	Kurtosis
Diameter of outer tube	*D* (mm)	Input	86	219	137	45	0.63	-0.85
Thickness of outer tube	*t* (mm)	Input	1.6	6	2.92	1.12	1.17	0.86
Column length	*L* (mm)	Input	188	660	396	141	0.32	-0.73
Yield strength of outer tube	*f*_*y*_ (mm)	Input	215	456	301	65	0.29	-1.16
Concrete strength	${f}_{c}{\prime}$(MPa)	Input	3.57	85.5	26.9	17	1.1	1.15
Rubber replacement ratio	*R*	Input	0	0.75	0.15	0.13	1.37	2.95
Diameter to thickness ratio	*D/t*	Input	23.8	82.5	49.3	13.39	0.23	-0.02
Length to diameter ratio	*L/D*	Input	1.97	4.00	2.94	0.77	0.42	-0.94
Axial load	*P*_*u*_	–-	162	3310	1047	764	1.18	0.5
Strength index	*p*_*si*_	Output	0.84	2.15	1.33	0.24	0.75	0.52

### ML algorithms

In this study, six typical ML models are selected to predict the axial strength of CS-RCFST columns, including symbolic regression (SR)^19,33^, Gaussian process (GPR)^10^, light gradient-boosting machine (LightGBM)^34^, random forests (RF)^35^, categorical boosting (CatBoost)^36^ and extreme gradient boosting (XGBoost)^37^. The predictive performances of these models are then evaluated and compared with two different code standards. Ensemble learning generally exhibits higher accuracy and stability than individual models^37^.

Random forests, proposed by Breiman^35^, fall under the category of ensemble learning based on bagging, which utilises bagging sampling to create a subset for training weak learners (such as decision trees) and makes decisions on regression or classification tasks through averaging or voting. Several crucial parameters, including the number of trees, the maximum number of features, and the maximum depth of trees, significantly impact the training results. On the other hand, CatBoost, LightGBM, and XGBoost are all part of ensemble learning based on boosting, which combines weak learners into a strong one through an iterative process^38^. CatBoost excels in handling categorical features, eliminating the need for preprocessing non-numerical features^36^. It solves the problem of gradient bias and enhances the generalisation ability by employing unbiased boosting techniques with categorical features. LightGBM^34^ uses a histogram-based approach for splitting, while XGBoost^37^ utilises a level-wise depth-first approach, which displays faster training times and better handling of large databases with LightGBM compared to XGBoost.

Furthermore, the min–max scaling technique is employed for data normalisation to alleviate the adverse effects of multidimensionality. Following normalisation, the datasets are divided into two subsets for training and testing purposes, with 80% of the original dataset randomly allocated for training and the remaining 20% reserved for testing.

The effectiveness of most ML algorithms relies largely on their hyperparameters, which are predefined prior to model training. Hyperparameters tuning involves trying various sets of hyperparameters and selecting the combination that results in the best performance with the validation data. Traditional techniques like grid search (GS) and random search (RS) have proved exhaustive and time-consuming, particularly for models with numerous hyperparameters and extensive search spaces. Conversely, other sophisticated models, such as Bayesian Optimization (BO) models, employ surrogate functions, i.e., Gaussian processes and tree-structured Parzen estimators (TPE)^39^, to guide the selection of the next hyperparameter combination based on the performance history of previously tested combinations. This approach minimises redundant evaluations, enabling BO to converge on the optimal hyperparameter combination in fewer iterations compared to GS and RS methods^39^. In this study, the TPE model^39^ is utilised to optimise the introduced ML models due to its superior robustness compared to other surrogate functions^39^.

The expected improvement (EI) used by the TPE model is defined in Eq. (11) . It constructs a probability model for the objective function and uses it to choose the best promising hyperparameters in the true objective function^40^:

2 $$E{I}_{{s}^{*}}\left(z\right)=\frac{\text{constant w}.\text{r}.\text{t }\left(z\right)}{\gamma +\left(1-\gamma \right)\frac{g\left(z\right)}{l\left(z\right)}}$$

where z is the hyperparameter combination chosen from the search space, and s* is a threshold chosen to be some quantile γ of the observed s values, so that $p\left(s<{s}^{*}\right)=\gamma$. Additionally, $l\left(z\right)$ and $g\left(z\right)$ correspond to two distinct distributions: one where the objective function values are below the threshold, l(z), and another where the values exceed the threshold, g(z). To maximize EI, TPE focuses on drawing samples of hyperparameters with the maximum l(z)/g(z) ratios, from Eq. (11). Finally, cross-validation was applied to assess the introduced models’ effectiveness, avoid overfitting, and obtain accurate predictions for the testing data. The hyperparameters optimized for introduced ML models are summarized in Table 3.

**Table 3:** The optimal hyperparameters for ML models.

ML model	Optimal hyperparameters
CatBoost	iterations = 1166, learning_rate = 0.059, depth = 5, subsample = 0.203, colsample_bylevel = 0.22, min_data_in_leaf = 78
GPR	Kernel: Constant*RBF + Constant*Matern + Constant*WhiteKernel + Constant* RationalQuadratic, gpr.alpha = 0.002
RandomForest	random_state = 1000,n_estimators = 929, max_depth = 30, min_samples_leaf = 2, max_features = ‘log2’, bootstrap = False

### Symbolic regression and proposed equation

Symbolic regression (SR)^19,33^ is a genetic programming technique^12^ that aims to discover interpretable analytic formulas that best fit a given model. Through exploring a predefined extensive space of mathematical expressions and functions, SR aims to balance predictive accuracy and complexity using genetic programming techniques, such as natural selection and evolution principles. In this study, the PySR Python library^41^ is utilised to search for simple and interpretable expressions for the axial capacity of the CS-RCFST columns.

**Fig. 2:**
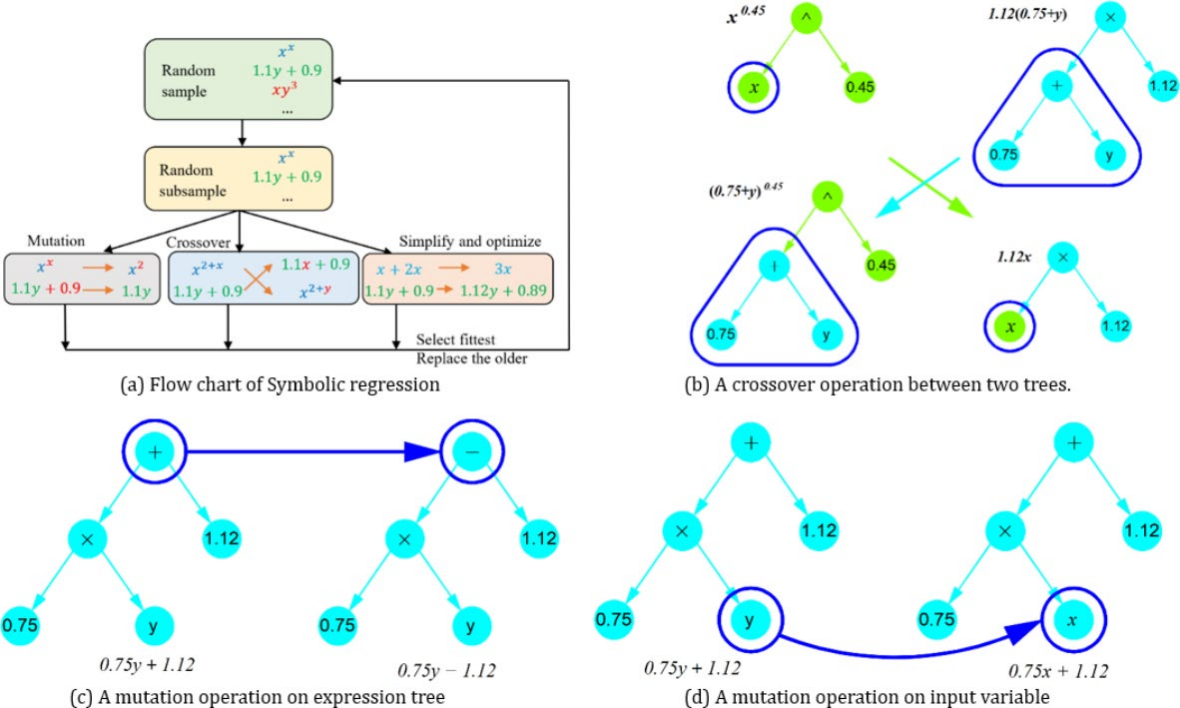
Symbolic regression.

The SR algorithm begins by creating an initial population comprising a random combination of operational symbols or functions (e.g., + , -, /, *, ^, etc.) and terminals, which include input variables and constants. This procedure generates a tree-like expression for each individual within the population. Individuals are then probabilistically chosen, with preference for those performing best. The fundamental steps of the SR algorithm are depicted in Fig. 2a. The selected individuals undergo mutation (Fig. 2b,c) or crossover (Fig. 2d) to generate a new generation of populations. Table 4 provides an overview of the SR parameters utilised in expression generation.

**Table 4:** The parameters of the SR model used in generating expressions.

Parameters	Value	Parameters	Value
Number of generations	200	Allowed Binary operators	+ , *, ^, /
Total number of populations	50	Loss function	Mean Absolute Error
Population size	20	Constraints	{‘^’:(–1,10)}^(a)^
Maximum length of expressions (total number of nodes)	20	Nested constraints	‘^’:{‘^’:0,’/’:1}, ‘/’:{‘/’:0,’^’:1}^(b)^
Parsimony (factor controls the expression complexity)	0.02	model_selection	Accuracy

^(a)^The constraint ‘^’:(–1,10) says that power laws can have any complexity in the left argument, but only 10 complexity (nodes) in the right argument.

^(b)^The nested constraints specify how many times a combination of operators can be nested. The constraint ‘/’:{‘/’:0,‘^’:1} indicates that ‘/’ may never appear within ‘/’, but ‘^’ can be nested once in ‘/’.

Choosing the optimal expression involves multiple iterations and a comprehensive exploration for each iteration. These iterations encompass trying various custom functions, a diverse set of operators, and extensive combinations of input features, which could potentially affect the axial strength of CS-RCFST columns^17^. The equation derived from each iteration undergoes thorough evaluation and refinement. The selection criteria carefully weigh multiple factors, such as equation complexity, accuracy, and interpretability, ensuring a comprehensive assessment of each candidate equation. For the axial strength of CS-RCFST columns, the following equation is derived:

3 $${P}_{u}={A}_{s}{f}_{y}+{A}_{c}{f}_{c}\left[0.9-0.81R+\frac{1.3}{\left(L/D\right)}+{\left(0.83\right)}^{{f}_{c}{\prime}}{\left({A}_{c}{f}_{c}{\prime}\right)}^{\alpha }\right],$$

where $\alpha =\frac{2{f}_{c}{\prime}}{{f}_{y}}+\frac{4.8}{\left(D/t\right)}$. From the above equation, the axial strength of CS-RCFST columns consists of two parts: the outer steel tube strength and concrete axial strength, while the bracket term defines the interaction between them. From Eq. (2), it is evident that reducing $L/D$, $D/t$, and $R\%$ ratios will enhance the columns axial capacity. These observations align well with the mechanical behaviour and experimental results of CS-RCFST columns^15,19–29^. Compared with the current code standards, i.e., AISC360^42^ and EC4^42^, as outlined in Table 5, the developed expression establishes a simple and robust framework for predicting the axial strength of CS-RCFST columns with meaningful physical interpretations.

**Table 5:** Code standards compared to the proposed equation for the axial compression capacity of the CS-RCFST columns.

Code Standard	Formulas
EC4^43^	${N}^{EC}={\eta }_{a}{f}_{y}{A}_{s}+\left(1+{\eta }_{c}\frac{t}{D}\frac{{f}_{y}}{{f}_{c}{\prime}}\right){f}_{c}{\prime}{A}_{c},$ ${\eta }_{a}=0.25\left(3+2\overline{\lambda }\right)\le 1.0, {\eta }_{c}=4.9-18.5\overline{\lambda }+17{\overline{\lambda }}^{2}\ge 0,$ $\overline{\lambda }=\sqrt{\frac{{N}_{pl}}{{N}_{cr}}}\le 0.5, {N}_{pl}={f}_{y}{A}_{s}+0.85{f}_{c}{\prime}{A}_{c},{N}_{cr}=\frac{{\pi }^{2}\left(E{I}_{eff}\right)}{{L}^{2}},E{I}_{eff}={E}_{s}{I}_{s}+0.6{E}_{c}{I}_{c}, {\lambda }_{max}=0.1\frac{{E}_{s}}{{f}_{y}}$
AISC360^42^	$${N}^{AISC}=\left\{\begin{array}{c}{f}_{y}{A}_{s}+0.95{f}_{c}{\prime}{A}_{c}, \lambda <{\lambda }_{p}\\ {N}_{pl}-\left(0.25{f}_{c}{\prime}{A}_{c}\right){\left(\frac{\lambda -{\lambda }_{p}}{{\lambda }_{r}-{\lambda }_{p}}\right)}^{2}, {\lambda }_{p}<\lambda <{\lambda }_{r}\\ \frac{0.72{f}_{y}{A}_{s}}{{\left(\lambda \frac{{f}_{y}}{{E}_{s}}\right)}^{0.2}}+0.7{f}_{c}{\prime}{A}_{c}, \lambda \ge {\lambda }_{r}\end{array}\right. \lambda =\frac{D}{t}, {\lambda }_{p}=0.15\frac{{E}_{s}}{{f}_{y}},{\lambda }_{r}=0.19\frac{{E}_{s}}{{f}_{y}}$$
Current study	${N}^{prop}={A}_{s}{f}_{y}+{A}_{c}{f}_{c}{\prime}\left[0.9-0.81R+\frac{1.3}{\left(L/D\right)}+{\left(0.83\right)}^{{f}_{c}{\prime}}{\left({A}_{c}{f}_{c}{\prime}\right)}^{\alpha }\right], \alpha =\frac{2{f}_{c}{\prime}}{{f}_{y}}+\frac{4.8}{\left(D/t\right)}$

where $\overline{\lambda }$ and $\lambda$ are the global and local buckling factors, respectively, $E{I}_{eff}$ is the flexural stiffeness, and ${\text{N}}_{\text{cr}}$ is Euler buckling load.

### Performance and results of ML models

**Fig. 3:**
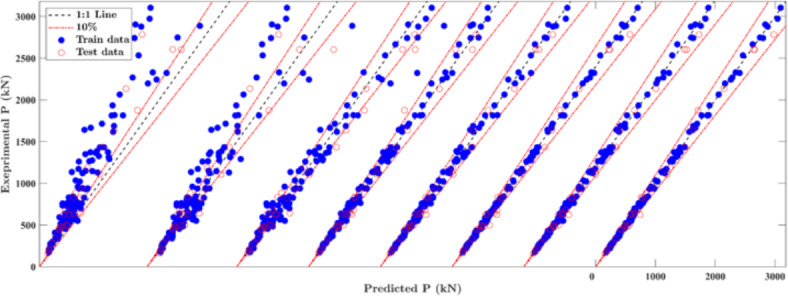
Comparison between established ML models and design standards for training and testing datasets.

In this section, the performance of the established ML models is compared with different code standards, i.e., AISC360^42^ and EC4^43^. The details of established developed ML models are provided in supplementary data, including hyperparameter tuning and results. Figure 3 illustrates scatter plots depicting the relationship between experimental and predicted results across different ML models. As observed, the data points tight closely around the diagonal line for most of the developed ML models, indicating strong alignment between model predictions and test results. The CATBoost, RF and GPR models exhibited outstanding accuracy, surpassing the remaining ML models and current design standards. The CATBoost model demonstrated the best prediction accuracy and generalisation ability, outperforming other ML models. Table 6 highlights evolution metrics used to assess the performance of the implemented models, including coefficient of determination (*R*^2^), mean (*μ*), coefficient of variance (CoV), mean absolute percentage error (MAPE%), root mean squared error (RMSE), and a20-index, expressed as follows:

4 $${R}^{2}=1-\frac{\sum_{i=1}^{n}{\left({\widehat{y}}_{i}-{y}_{i}\right)}^{2}}{\sum_{i=1}^{n}{\left({y}_{i}-\overline{y }\right)}^{2}}, \mu =\frac{1}{n}\sum_{i=1}^{n}\frac{{y}_{i}}{{\widehat{y}}_{i}}, MAPE\%=\frac{100\%}{n}\sum_{i=1}^{n}\left|\frac{{y}_{i}}{{\widehat{y}}_{i}}-1\right|, RMSE=\sqrt{\frac{1}{n}\sum_{i=1}^{n}{\left({\widehat{y}}_{i}-{y}_{i}\right)}^{2}}$$

where ${\widehat{y}}_{i}$ and ${y}_{i}$ represent the predictions and actual output values of the *i-*th specimen, respectively, $\overline{y }$ is the mean value of actual observations, and *n* is the number of samples in the database. The a20-index^44^ gives the ratio of specimens with ${y}_{i}/{\widehat{y}}_{i}$ ratio lying within the interval of 0.80 to 1.20.

**Table 6:** Comparison of the developed ML models.

Metrics	Training data			Testing data			All data					
	CatB	RF	GPR	CatB	RF	GPR	CatB	RF	GPR	Prop. Eqn	AISC^42^	EC4^43^
Mean $\mu$	0.999	0.999	0.999	1.001	0.984	0.985	1.00	0.996	0.996	0.99	1.363	1.053
CoV	0.03	0.039	0.029	0.061	0.057	0.074	0.039	0.043	0.042	0.132	0.322	0.187
R^2^	0.999	0.997	0.999	0.993	0.994	0.993	0.998	0.997	0.998	0.967	0.706	0.891
MAPE%	2.365	2.963	2.064	4.993	3.993	5.528	2.891	3.169	2.756	9.995	36.65	15.71
RMSE(kN)	28.2	38.8	23.5	63.8	58.2	64	38.1	43.3	35.5	137.7	412.8	251.9
a20-index	1.00	1.00	1.00	1.00	1.00	1.00	1.00	1.00	1.00	0.883	0.297	0.703

As outlined in Table 6, all introduced ML models display mean *μ*, *R*^2^, and a20-index values close to 1.0 and small values for error indices, i.e., CoV, MAPE%, and RMSE. The MAPE% values for the CATB model are 2.37% and 4.99% in the training and testing sets, respectively, which reach the lowest values compared to other models. Similarly, those of the RF model are 2.96% and 3.99%, and those of the GPR model are 2.06% and 5.53%, indicating the high accuracy of the developed models. Although the GPR model exhibits slightly higher errors for the testing data, achieving a CoV of 0.074 compared to the training set error with a CoV of 0.029, its overall performance, as measured by remaining evolution metrics, is comparable to that of other ML models. In contrast, the RF model demonstrates consistent behaviour across both training and testing results, displaying similar evolution metrics for both sets, which indicate minimal overfitting tendencies. Such evaluation metrics reveal that the CATB model introduces the best prediction accuracy and predictive balance between the training and testing sets.

While CATB, RF, and GPR models exhibit superior results, deriving an explicit design formula from these models is challenging. The black-box and difficult-to-interpret nature of these models hinders their practical implementation in engineering design. Therefore, this study tackles this challenge by introducing a straightforward and practical explicit design formula through the SR technique. As shown in Table 6, the proposed equation yields *μ* values of 0.99, *R*^2^ values of 0.967, and CoV value of 0.132 for the axial load capacity of CS-RCFST columns. Despite their slightly lower accuracy compared to the introduced ML models, these SR-derived formulas are more accessible and easier to interpret, encouraging their practical usage in engineering applications.

This section compares the results of the developed expression with those of two different design standard codes, i.e., AISC360^41^ and EC4^42^, for performance evaluation. The scatter plots in Fig. 3 illustrate the relationship between experimental and predicted results for the proposed expression and the two code standards. AISC360 results exhibit an over-diagonal-skewed distribution with (μ, CoV) values of (1.363, 0.322), indicating that it tends toward conservative prediction. The EC4 standard, while displaying a mean value near unity with (μ, CoV) values of (1.053, 0.187), yields conservative results for a significant portion of the data, as depicted in Fig. 3. On the other hand, the proposed equation demonstrates concentrated test-to-prediction ratios around unity, with (μ, CoV) values of (0.99, 0.132), which shows that the introduced expression performs well in terms of predictive stability and robustness compared to the present design standards. Additionally, when comparing the results of code standards with those of ML models, the CATBoost model demonstrates superior performance with (μ, CoV) values of (1.00, 0.039), highlighting its outstanding efficacy in leveraging ML techniques for predicting the axial strength of CS-RCFST columns.

**Fig. 4:**
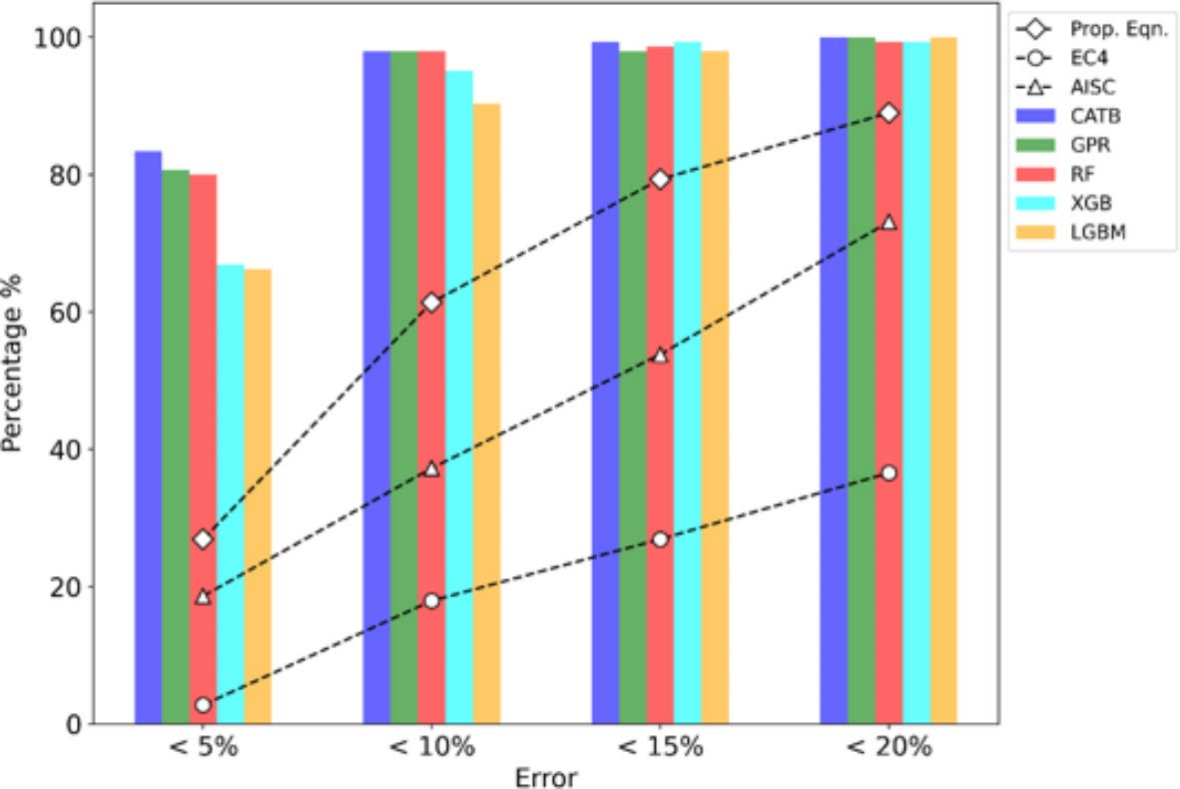
Prediction errors of design standards and established ML models.

Figure 4 illustrates the prediction errors of both design standards and the developed ML models. The CATB, RF, and GPR models demonstrate precision, with over 98% of test samples falling within the 10% error range. Meanwhile, the developed expression exhibits 61% of test samples within the same error range. As noticed, AISC360 and EC4 provisions perform less effectively, capturing only 18% and 37% of test samples within the 10% error range, respectively. These results highlight the superior accuracy of most ML models, particularly CATB, RF, and GPR, in predicting the axial strength of CS-RCFST columns compared to current design standards. Furthermore, the proposed equation displays satisfactory accuracy compared to the black-box ML models discussed earlier.

### Reliability analysis

Despite the extensive knowledge available on the axial compressive behaviour of CS-RCFST columns, there is a lack of clear standards to ensure their safe design. Applying existing design resistance factors from current code standards without modifications may compromise the safety of CS-RCFST columns, mainly due to significant differences in strength uncertainties associated with utilising rubber aggregates instead of conventional aggregates in concrete construction. Therefore, this section involves identifying the statistical properties associated with the compressive strength of rubber aggregate concrete (RBAC). Moreover, it introduces the required resistance design factors *ϕ* aligned with the target reliability recommended by code standards for the proposed equation, the CATB model, and two existing code standards, including AISC360^42^ or EC2^43^.

The limit state function *g* of axial strength of CS-RCFST columns^45^ can be defined as:

5 $$g=R-Q={\theta }_{\text{R}}{R}_{uc}-\left({D}_{n}+{L}_{n}\right)$$

where *R* is the random values of axial strength of CS-RCFST columns, defined as the predicted axial capacity (*R*_*uc*_) multiplied by the test-to-prediction ratio ${\theta }_{R}$, and *Q* is the random values of load effect, including the dead load (*D*_*n*_) and live load (*L*_*n*_). The value *R*_*uc*_ is calculated for each column from Table 5 with the partial resistance factors taken as unity, and using the random values of design variables given in Table 7. Using the distribution fit tool in Matlab, it was found that ${\theta }_{\text{R}}$ ratio is best fitted with lognormal distribution with mean and variance corresponding to each code standard, as indicated in Table 8. The nominal values *D*_*n*_ and *L*_*n*_ can be computed from the design resistance *R*_*d*_ for a given live-to-dead load ratio (*L*_*n*_/*D*_*n*_) as follows:

6 $${R}_{d}\left(\frac{{f}_{ck}}{{\gamma }_{c}},\frac{{f}_{y}}{{\gamma }_{s}}\right) or \phi {R}_{n}\left({f}_{ck},{f}_{y}\right)={S}_{d}\left(i.e.{\gamma }_{D}{D}_{n}+{\gamma }_{L}{L}_{n}\right)$$

7 $${D}_{n}=\frac{{R}_{d} or \phi {R}_{n}}{{\gamma }_{D}+{\gamma }_{L}.k}$$

where k is the live-to-dead load ratio (*L*_*n*_/*D*_*n*_), the reduced designed resistance (*R*_*d*_) is extracted from dividing the characteristic strength of concrete and steel materials (${f}_{ck}$ and *f*_*y*_) by the material partial factors (*γ*_*c*_ and *γ*_*s*_)^42^ or multiplying the nominal resistance (*R*_*n*_) by a strength reduction factor (*ϕ*)^43^, and then *R*_*d*_ is balanced by the enlarged designed load effect (*S*_*d*_) to ensure a suitable safety margin. *S*_*d*_ is obtained by multiplying the nominal load values, including dead and live loads (*D*_*n*_ and *L*_*n*_), by, respectively, partial load factors (*γ*_*D*_ and *γ*_*L*_) and then combining them linearly. These partial factors are summarised in Table 7 for each code standard.

**Table 7:** Statistical properties of random variables for CS-RCFST columns.

Properties	Variables	Mean	CoV	Distribution	Space	Ref
Geometry	D	1.0	0.005	Lognormal	*D/t* = {20,40,60,80}	^48,49^
	t	0.99	0.025	Lognormal	*t* = {1.0,3.0,6.0}	^48,49^
	*L*	1.0	-	Deterministic	*L/D* = {2.0, 4.0}	
Material	${f}_{c}{\prime}$*(R* = *0)*	1.08	0.15	Lognormal	${f}_{c}{\prime}$= {20,30,40,60,90} *R* = {0, 0.1,0.2,0.3}	^23,49,50^ and Table 7
	${f}_{c}{\prime}$*(R* = *0.10)*	1.08	0.228			
	${f}_{c}{\prime}$*(R* = *0.20)*	1.08	0.297			
	${f}_{c}{\prime}$*(R* = *0.30)*	1.08	0.366			
	${f}_{y}$< 355 MPa	1.25	0.055	Lognormal	${f}_{y}$= {240,360,430,520}	^48,51^
	${f}_{y}$< 420 MPa	1.2	0.050			
	${f}_{y}$< 460 MPa	1.15	0.045			
	${f}_{y}$≥ 460 MPa	1.1	0.035			
Load	*k* (load ratio)	k	-	Deterministic	*k* = {0.5,1.25,2.0}	
	*D* (dead load)	1.0	0.1	Normal	-	^21^
	*L* (live load) AISC360	1.0	0.25	Gumbel	-	^21^
	*L* (live load) EC4	0.6	0.35	Gumbel	–	^22^

**Table 8:** Load factors, the test-to-prediction ratio ${\theta }_{R}$ distributions, recommended strength reduction factor ϕ for different rubber replacement ratios *R*.

		AISC360^42^	EC4^43^	CATB	Prop. Eqn
Load factor $\gamma$	Dead load ${\gamma }_{D}$ Live load ${\gamma }_{L}$	1.2 1.6	1.35 1.5	1.2* 1.6*	1.2* 1.6*
Best-fit distribution ${\theta }_{\text{R}}$ (Table 5)		Lognormal	Lognormal	Lognormal	Lognormal
	Mean (Table 5)	1.363	1.053	1.00	0.99
	CoV (Table 5)	0.322	0.187	0.039	0.132
Target reliability $\beta$		3.0	3.8	3.0*	3.0*
Evaluated strength reduction factor $\phi$ for the target reliability index	*R* = 0.0	0.66	0.75	1.0 (*β* = 4.63)	0.76
	*R* = 0.1	0.63	0.72	1.0 (*β* = 3.79)	0.72
	*R* = 0.2	0.61	0.69	1.0 (*β* = 3.27)	0.68
	*R* = 0.3	0.58	0.66	0.98 (*β* = 3.0)	0.64

*: Load factors and target reliability $\beta$ are assumed to be identical to those of AISC360.

The safety level of structures can be measured by the reliability index *β*, a factor related to the failure probability *P*_*f,*_ as follows^46^:

8 $$\beta ={\Phi }^{-1}\left({P}_{f}\right)$$

where Φ is the standard cumulative distribution function. Monte Carlo simulation (MCS) is employed to determine the reliability index due to its simplicity, insensitivity to problem dimensions, and satisfactory accuracy^47^. In MCS, the failure probability can be calculated as

9 $${P}_{f}=\frac{{N}_{fail}}{N}$$

where *N* and *N*_*fail*_ are the total number of simulations and the number of failed simulations (when the limit state function is violated, i.e. g ≤ 0), respectively. The target safety level stipulated by AISC360^42^ and EC4^43^ provisions for the axial strength of CS-RCFST columns are 3.0 and 3.8, respectively. The accuracy of MCS is dependent on the number of samples *N*. The number of samples *N* used in this study for achieving a reliability index β equal to 3.8 with acceptable accuracy (CoV of 5%) is 5,528,430^20^.

### Random variables

To accurately predict the reliability index of the design codes, the uncertainty or randomness of all input variables, including material geometry and loads, should be considered^45^. Eight random variables are considered in this study, and the statistical properties are summarised in Table 7. The random numbers of variable inputs are generated with continuous variations stochastically chosen from their respective distribution functions (Table 7) and drawn from a wide range of geometric and geometry parameters of CS-RCFST column configurations. They include five values of concrete compressive strength $f_{c}^{\prime }$ = {20, 30, 40, 60, 90} MPa with four replacement ratio *R* = {0, 0.1, 0.2, 0.3}, three values of tube thickness *t* = {1.0, 3.0, 6.0} mm, four values of diameter-to-thickness ratio *D/t* = {20, 40, 60, 80}, two values for Length-to-diameter ratio *L/D* = {2.0, 4.0}, four values of steel yield stress *f*_*y*_ = {240,360,430,520} MPa, three ratios of *L*_*n*_/*D*_*n*_ = {0.5, 1.25, 2.0}. In total, there are 5 × 4 × 3 × 4 × 2 × 4 × 3 = 5760 column configurations considered for each considered model. The statistical distribution of the dead load *D*_*n*_ is derived from reference^21^, where the characteristic value is considered equivalent to the mean value. Regarding the live load *L*_*n*_, two distinct approaches are employed by the US and European standards to determine its characteristic value. In US standards, this value is established as the mean of the 50-year maxima^21^. Conversely, European standards define the characteristic value of the live load as the 98th percentile of the annual maximum loads^22^, resulting in a value of 0.6. It is important to highlight that the target reliability index for columns designed according to EC4 (β = 3.8) significantly exceeds those designed using AISC360 (e.g., β = 3.0). This variance primarily stems from the utilisation of the live load characteristic value of 0.6 in Eurocode standards^22^ (refer to Table 7), which is lower than the mean value of 1.0 employed in American standards^21^.

Despite the apparent safety of the previously mentioned code standards in predicting the axial strength of CS-RCFST columns, there is a relatively large coefficient of variation (COV) expected in the axial capacity due to the inferior mechanical properties of concrete with rubber aggregate (RBAC) when using equivalent concrete compressive strength. This implies that if the strength of RBAC is equivalent to that of concrete with normal aggregate (NAC), the axial strength of the corresponding columns should be similar, while there is relatively high uncertainty regarding the reliability of predictions. Therefore, the existing design resistance factors for NAC in current code standards must be further adjusted when using RBAC.

In this study, the authors used the mean and CoV of the concrete strength $f_{c}^{\prime }$ for NAC, obtained from Bartlett and Macgregor^23^, which are reported as (1.08, 0.15) respectively. To quantify the uncertainty in concrete compressive strength resulting from using rubber aggregate (RA), CoV values from various experimental studies involving RBAC at three different replacement ratios {0.1,0.2,0.3} are collected and corrected to be consistent with site conditions (see Table 9). A correction factor *f*, defined as the ratio of CoV of RBAC for different replacement ratios to that of NAC, is computed to reflect the effect of incorporating rubber aggregate. As outlined in Table 9, the CoV of concrete strength $f_{c}^{\prime }$ for NAC is finally adjusted by multiplying its value by the average correction factor to address uncertainties associated with using RBAC.

**Table 9:** Coefficient of variance for statistical properties of rubber aggregate concrete.

	Reported CoV				CoV/CoV_(R=0.0)_		
Ref	*R* = 0.0	*R* = 0.1	*R* = 0.2	*R* = 0.3	*R* = 0.1	*R* = 0.2	*R* = 0.3
^52^	0.108	0.135	0.162	0.189	1.25	1.50	1.74
^53^	0.041	0.043	0.046	0.049	1.07	1.14	1.20
^54^	0.059	0.104	0.15	0.195	1.77	2.53	3.30
^55^	0.079	0.108	0.138	0.167	1.38	1.75	2.13
^56^	0.03	0.047	0.064	0.081	1.58	2.15	2.73
^57^	0.05	0.125	0.18	0.235	2.50	3.60	4.70
^2^	0.089	0.098	0.107	0.116	1.10	1.20	1.30
Average magnification factor (*f*)					1.52	1.98	2.44
Prop. CoV = CoV_NAC_$\times$(*f*) = 0.15^i^$\times$(*f*)					0.228	0.297	0.366

i: where CoV_NAC_ is the site CoV for concrete with normal aggregate (NAC). Its value is 0.15, as documented in references^49,50^.

### Reliability analysis results

**Fig. 5:**
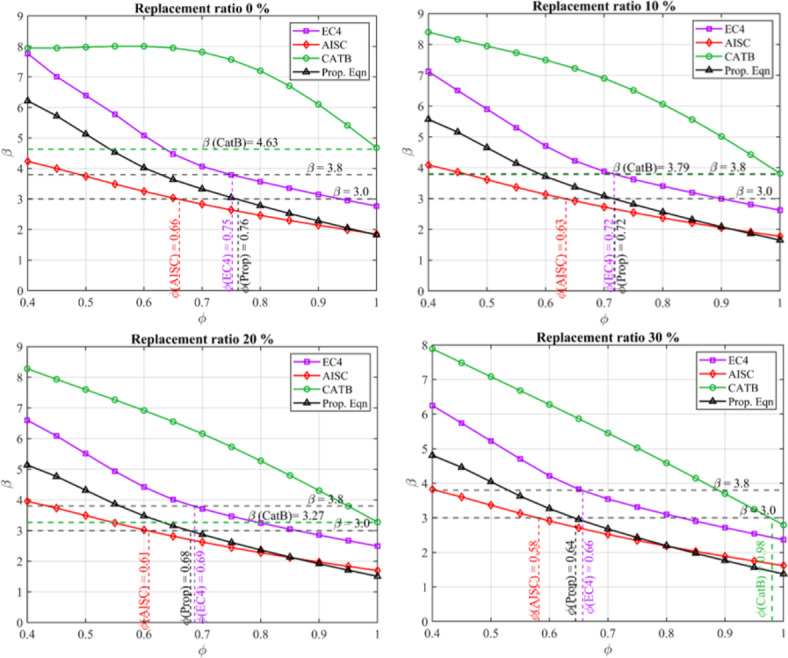
Strength reduction factor *ϕ* for CS-RCFST columns designed by the proposed equation, CatBoost model, AISC360 and EC4 for different rubber replacement ratios.

Figure 5 and Table 8 display strength reduction factor *ϕ* for CS-RCFST columns designed by the proposed equation, CatBoost model, AISC360 and EC4 for different rubber replacement ratios. In Fig. 5, for each reduction factors (*ϕ*), incremented by 0.05, the average beta value was calculated across all 5760 column configurations. Specifically, for each configuration, random variables were generated with means corresponding to the configuration’s parameters and coefficients of variation (CoV) or standard deviation as specified in Table 7. The beta values were then computed for each of these 5760 configurations, and an average beta value was determined, providing a representative measure across the entire column configurations. At a reliability index value of 3.0, the proposed equation exhibits strength reduction factors (*ϕ*) of {0.76, 0.72, 0.68, 0.64} for replacement ratios of {0,0.1,0.2,0.3}, respectively, while AISC360 and EC4 standards display {0.66,0.63,0.61,0.58} and {0.75,0.72,0.69,0.66} at reliability index values of 3.0 and 3.80, respectively, for the same replacement ratios. As observed in Table 8, the strength reduction factor decreases with increasing the replacement ratio due to the increased uncertainties resulting from aggregate replacement with rubber. Furthermore, the developed expression is more reliable than AISC360 results for the same reliability index and live load statistical distribution. Moreover, the proposed equation displays a mean value *μ* of 0.99 compared to that of AISC360 (*μ* = 1.363) and EC4 (*μ* = 1.053), which can potentially introduce economic structural members without compromising structural safety. Moreover, Fig. 5 reveals that the CATB model, when used with *ϕ* = *1.0*, can achieve a relatively high-reliability index of {4.63, 3.79, 3.27, 2.98} for replacement ratios of {0,0.1,0.2,0.3}, respectively. This high-reliability index is attributed to the low CoV error metric of the CATB model compared to other models, as previously outlined in Table 6. These findings emphasis the high reliability of ML models to improve the predictive accuracy for the axial strength of CS-RCFST columns.

### Limitations

This section discusses the limitations of the machine learning models and reliability analysis, particularly their dependence on the data used for their development. These models are specifically tailored to the geometries and material properties within the dataset, as detailed in Table 1. For instance, the D/t ratios range from 23.8 to 83, and the L/D ratios are confined to short columns with values between 2.0 and 4.0. Similarly, the concrete compressive strengths and steel yield strengths are also limited to specific ranges. Additionally, the diameters and thicknesses are restricted to only 219 mm and 6 mm, respectively, which may not encompass all real-world scenarios. Therefore, applying these models and reliability analysis to columns with geometries or material properties outside these ranges could result in inaccurate predictions due to the extrapolation beyond the model’s trained data. Future work could focus on expanding the dataset to include a wider range of geometries and material properties to enhance the applicability of these models.

### Research significance

This study utilises machine learning (ML) algorithms for predicting the compressive strength of rubber aggregate concrete-filled steel tubular (CFST) circular stub columns. In addition, a reliability analysis is performed to evaluate the reliability of incorporating rubber aggregate concrete CFST columns.

## Conclusions

In conclusion, this study compiled a comprehensive database of 145 experimental tests for the axial strength of CS-RCFST columns from various research papers. It employed six machine learning models optimised using the Bayesian Optimization (BO) technique. In addition, a proposed design expression is introduced for designing CS-RCFST columns. Furthermore, a reliability analysis is performed to assess the impact of using RBAC on the axial strength of the CFST columns. From the evolution results, the following conclusions can be drawn:

- The CATBoost, RF and GPR models exhibited outstanding accuracy and stability, surpassing the current design standards. The CATBoost model demonstrated the best prediction accuracy and generalisation ability, outperforming other ML models.
- The introduced explicit design formula, derived through symbolic regression, stands out for its simplicity and robustness compared to the black-box ML models and current code standards.
- The code standards outlined in AISC360 and EC4, originally specified for normal CFST columns, demonstrate promising acceptable accuracy in predicting the axial load-carrying capacity of CS-RCFST columns. This is attributed to the fact that the influence of incorporating RBAC on the axial load-carrying capacity is primarily reflected in the concrete strength. However, their design strength factors should be adjusted to accommodate the increased uncertainties associated with replacing conventional aggregates with rubber.
- The reliability analysis has shown that the CATBoost model and developed expression surpassed code standards regarding reliability, accuracy, and economical design.

In summary, integrating the ML-based approach is promising for accurately predicting the axial strength of CS-RCFST columns, providing valuable insights for engineering applications.

## Electronic supplementary material

Below is the link to the electronic supplementary material.

Supplementary Material 1

## Data Availability

All data generated or analysed during this study are included in this published article and available in a public repository: https://github.com/kmegahed/CS-RCFST-columns.

## References

[1] Lu, S., Yang, J., Wang, J., Huang, L. & Wang, L. Behavior of steel tubed rubberized geopolymer concrete columns under axial compression: Experimental study and analytical modeling. *Eng. Struct.* **302**, 117389. 10.1016/j.engstruct.2023.117389 (2024).

[2] Duarte, A. P. C. et al. Finite element modelling of short steel tubes filled with rubberized concrete. *Compos. Struct.* **150**, 28–40. 10.1016/j.compstruct.2016.04.048 (2016).

[3] Xu, P. Z., Li, H. W., Yan, H. S. & Zhu, Y. G. Effect of rubber content on mechanical properties of round rubber concrete-filled steel tubular short columns. *Build. Struct.* **495**(3), 71–75 (2019).

[4] Nematzadeh, M., Karimi, A. & Gholampour, A. Pre- and post-heating behavior of concrete-filled steel tube stub columns containing steel fiber and tire rubber. *Structures* **27**(April), 2346–2364. 10.1016/j.istruc.2020.07.034 (2020).

[5] Nematzadeh, M., Memarzadeh, A. & Karimi, A. Post-fire elastic modulus of rubberized fiber-reinforced concrete-filled steel tubular stub columns: Experimental and theoretical study. *J. Constr. Steel Res.* **175**, 106310. 10.1016/j.jcsr.2020.106310 (2020).

[6] Elchalakani, M. et al. Experimental tests and design of rubberised concrete-filled double skin circular tubular short columns. *Structures* **15**(February), 196–210. 10.1016/j.istruc.2018.07.004 (2018).

[7] Abuzaid, O., Nabilah, A. B., Safiee, N. A. & Noor Azline, M. N. Rubberized concrete filled steel tube. *IOP Conf. Ser. Earth Environ. Sci.*10.1088/1755-1315/357/1/012014 (2019).

[8] Jafarifar, N., Bagheri Sabbagh, A. & Uchehara, I. Rubberised concrete confined with thin-walled steel profiles: A ductile composite for building structures. *Structures* **49**, 983–994. 10.1016/j.istruc.2023.01.134 (2023).

[9] Rezaeicherati, F. et al. Experimental study of post-fire bond behavior of concrete-filled stiffened steel tubes: A crucial aspect for composite structures. *Structures* **62**, 106203. 10.1016/j.istruc.2024.106203 (2024).

[10] Rasmussen, C. E., & Williams, C. K. I. *Gaussian Processes for Machine Learning*, vol. 1. Springer (2006).

[11] Megahed, K., Mahmoud, N. S. & Abd-Rabou, S. E. M. Application of machine learning models in the capacity prediction of RCFST columns. *Sci. Rep.* **13**(1), 20878. 10.1038/s41598-023-48044-1 (2023). 38012229 10.1038/s41598-023-48044-1 PMC10682462

[12] Goldberg, D. E. & Holland, J. H. Genetic algorithms and machine learning. *Mach. Learn.* **3**(2), 95–99. 10.1023/A:1022602019183 (1988).

[13] Güneyisi, E. M., Gültekin, A. & Mermerdaş, K. Ultimate capacity prediction of axially loaded CFST short columns. *Int. J. Steel Struct.* **16**(1), 99–114. 10.1007/s13296-016-3009-9 (2016).

[14] Hou, C. & Zhou, X. G. Strength prediction of circular CFST columns through advanced machine learning methods. *J. Build. Eng.* **51**, 104289. 10.1016/j.jobe.2022.104289 (2022).

[15] Ahmadi, M., Naderpour, H. & Kheyroddin, A. ANN model for predicting the compressive strength of circular steel-confined concrete. *Int. J. Civ. Eng.* **15**(2), 213–221. 10.1007/s40999-016-0096-0 (2017).

[16] Zarringol, M., Thai, H.-T., Thai, S. & Patel, V. Application of ANN to the design of CFST columns. *Structures* **28**, 2203–2220. 10.1016/j.istruc.2020.10.048 (2020).

[17] Megahed, K., Mahmoud, N. S. & Abd-Rabou, S. E. M. Prediction of the axial compression capacity of stub CFST columns using machine learning techniques. *Sci. Rep.* **14**(1), 2885. 10.1038/s41598-024-53352-1 (2024). 38311629 10.1038/s41598-024-53352-1 PMC10838919

[18] Chen, W., Xu, J., Li, Z., Huang, X. & Wu, Y. Load-carrying capacity of circular recycled aggregate concrete-filled steel tubular stub columns under axial compression: Reliability analysis and design factor calibration. *J. Build. Eng.* **66**, 105935. 10.1016/j.jobe.2023.105935 (2023).

[19] Koza, J. R. Genetic programming as a means for programming computers by natural selection. *Stat. Comput.* **4**(2), 87–112. 10.1007/BF00175355 (1994).

[20] Soong, T. T. & Grigoriu, M. Random vibration of mechanical and structural systems. *NASA STI/Recon Tech. Rep. A* **93**, 14690 (1993).

[21] Galambos, T. V. Load and resistance factor design. *Eng. J.* **18**(3), 74–82 (1981).

[22] E. Commission *et al.*, *Reliability of structural members designed with the Eurocodes NDPs selected by EU and EFTA Member States*. Publications Office (2018).

[23] Bartlett, F. M. & MacGregor, J. G. Statistical analysis of the compressive strength of concrete in structures. *Mater. J.* **93**(2), 158–168 (1996).

[24] Yang, W., Jiao, Y., & Liping, F. Study on Mechanical characteristics of axial compression of short column of confined rubber concrete filled steel tube. *J. Shenyang Jianzhu Univ.* **36**(4) (2020).

[25] Haoran, X., Niandong, M., & Xinran, Y. Experimental study on the axial compression performance of steel tube rubber aggregate concrete short columns. *Silic. Bull.* (2021).

[26] Liang, J. F., Jiang, L. Z., Wu, H. Y. & Gu, L. S. Experimental study on mechanical properties of rubber concrete filled steel tube column under axial compression. *J. Guangxi Univ.* **42**(1), 134–141 (2017).

[27] Mohanraj, E. K., Malathy, R. & Ravisankar, K. L. Utilization of industrial waste materials in concrete-filled steel tubular columns. *Rev. Mater.*10.1590/S1517-707620220002.1388 (2022).

[28] Elshazly, F. A., Mustafa, S. A. A. & Fawzy, H. M. Analysis of strengthened short deficient rubberized concrete-filled steel tubular columns. *Frat. ed Integrita Strutt.* **15**(55), 1–19. 10.3221/IGF-ESIS.55.01 (2021).

[29] Ahmad, S., Kumar, K. & Kumar, A. Axial behaviour of steel tubes filled with concrete incorporating high-volume rubber. *Innov. Infrastruct. Solut.* **7**(2), 1–11. 10.1007/s41062-022-00739-6 (2022).

[30] Hossain, K. M. A. & Chu, K. Confinement of six different concretes in CFST columns having different shapes and slenderness. *Int. J. Adv. Struct. Eng.* **11**(2), 255–270. 10.1007/s40091-019-0228-2 (2019).

[31] Mujdeci, A., Bompa, D. V. & Elghazouli, A. Y. Confinement effects for rubberised concrete in tubular steel cross-sections under combined loading. *Arch. Civ. Mech. Eng.* **21**(2), 1–20. 10.1007/s43452-021-00204-8 (2021).

[32] Deng, Y., Liang, J. F. & Li, W. Axial performance of steel fiber-reinforced rubberized concrete-filled circular tubular columns. *Adv. Mater. Sci. Eng.*10.1155/2021/6678802 (2021).

[33] Udrescu, S.-M. & Tegmark, M. AI Feynman: A physics-inspired method for symbolic regression. *Sci. Adv.* **6**(16), eaay2631 (2020). 32426452 10.1126/sciadv.aay2631 PMC7159912

[34] Ke, G. *et al.* LightGBM: A highly efficient gradient boosting decision tree, in *Advances in Neural Information Processing Systems* (2017). https://proceedings.neurips.cc/paper_files/paper/2017/file/6449f44a102fde848669bdd9eb6b76fa-Paper.pdf.

[35] Breiman, L. Random forests. *Mach. Learn.* **45**(1), 5–32. 10.1023/A:1010933404324 (2001).

[36] Dorogush, A. V., Ershov, V., & Gulin, A. CatBoost: Gradient boosting with categorical features support. CoRR. http://arxiv.org/abs/1810.11363.

[37] Chen, T., & Guestrin, C. XGBoost: A scalable tree boosting system, in *Proceedings of the 22nd ACM SIGKDD International Conference on Knowledge Discovery and Data Mining*, 2016, pp. 785–794. 10.1145/2939672.2939785.

[38] Schapire, R. E. The strength of weak learnability. *Mach. Learn.* **5**(2), 197–227. 10.1007/BF00116037 (1990).

[39] Yang, L. & Shami, A. On hyperparameter optimization of machine learning algorithms: Theory and practice. *Neurocomputing* **415**, 295–316. 10.1016/j.neucom.2020.07.061 (2020).

[40] Bergstra, J., Bardenet, R., Bengio, Y., & Kégl, B. Algorithms for hyper-parameter optimization, in *Advances in Neural Information Processing Systems* (2011). https://proceedings.neurips.cc/paper_files/paper/2011/file/86e8f7ab32cfd12577bc2619bc635690-Paper.pdf.

[41] Cranmer, M. Interpretable Machine Learning for Science with PySR and SymbolicRegression.jl (2023). http://arxiv.org/abs/2305.01582.

[42] AISC, “AISC 360-22 Specification for Structural Steel Buildings. *Am. Inst. Steel Constr.*, 780 (2022).

[43] Beng, S. H., & Park, S. EN 1994-Eurocode 4: Design of composite steel and concrete structures. *Retrieved May* **10**, 2022 (1994).

[44] Asteris, P. G. & Mokos, V. G. Concrete compressive strength using artificial neural networks. *Neural Comput. Appl.* **32**(15), 11807–11826. 10.1007/s00521-019-04663-2 (2020).

[45] Nasrollahzadeh, K. & Aghamohammadi, R. Reliability analysis of shear strength provisions for FRP-reinforced concrete beams. *Eng. Struct.* **176**, 785–800. 10.1016/j.engstruct.2018.09.016 (2018).

[46] Rackwitz, R. & Flessler, B. Structural reliability under combined random load sequences. *Comput. Struct.* **9**(5), 489–494. 10.1016/0045-7949(78)90046-9 (1978).

[47] Nowak, A. S., & Collins, K. R. *Reliability of Structures*. CRC Press (2012).

[48] da Silva, L. S., Marques, L., Tankova, T., Rebelo, C., Kuhlmann, U., & Kleiner, A. *Standardization of Safety Assessment Procedures across Brittle to Ductile Failure Modes (SAFEBRICTILE)*. Publications Office of the European Union (2017).

[49] Thai, H.-T. et al. Reliability considerations of modern design codes for CFST columns. *J. Constr. Steel Res.* **177**, 106482 (2021).

[50] Nowak, A. S. & Szerszen, M. M. Calibration of design code for buildings (ACI 318): Part 1—Statistical models for resistance. *ACI Struct. J.* **100**(3), 377–382 (2003).

[51] Knobloch, M. et al. Structural member stability verification in the new Part 1–1 of the second generation of Eurocode 3. *Steel Constr.* **13**(2), 98–113. 10.1002/stco.202000016 (2020).

[52] Park, Y., Abolmaali, A., Kim, Y. H. & Ghahremannejad, M. Compressive strength of fly ash-based geopolymer concrete with crumb rubber partially replacing sand. *Constr. Build. Mater.* **118**(2016), 43–51. 10.1016/j.conbuildmat.2016.05.001 (2016).

[53] Mohammadi, I. & Khabbaz, H. Shrinkage performance of Crumb Rubber Concrete (CRC) prepared by water-soaking treatment method for rigid pavements. *Cem. Concr. Compos.* **62**, 106–116. 10.1016/j.cemconcomp.2015.02.010 (2015).

[54] Grinys, A., Sivilevičius, H. & Daukšys, M. Tyre rubber additive effect on concrete mixture strength. *J. Civ. Eng. Manag.* **18**(3), 393–401. 10.3846/13923730.2012.693536 (2012).

[55] Choi, Y., Kim, I.-H., Lim, H.-J. & Cho, C.-G. Investigation of strength properties for concrete containing fine-rubber particles using UPV. *Materials (Basel)* **15**(10), 3452 (2022). 35629480 10.3390/ma15103452 PMC9145537

[56] Liu, L. et al. Evaluation of the compressive-strength reducing behavior of concrete containing rubber aggregate. *Clean. Mater.* **4**, 100057. 10.1016/j.clema.2022.100057 (2022).

[57] Roychand, R. et al. A comprehensive review on the mechanical properties of waste tire rubber concrete. *Constr. Build. Mater.* **237**, 117651. 10.1016/j.conbuildmat.2019.117651 (2020).

