# Supplementary material for: Circular rubber aggregate CFST stub columns under axial compression: prediction and reliability analysis
Source: Sci Rep. 2024 Oct 31;14:26245. doi: 10.1038/s41598-024-74990-5 (PMC11527877; doi:10.1038/s41598-024-74990-5)
Supplement: Supplementary file 1 — Supplementary Material 1 [file 41598_2024_74990_MOESM1_ESM.zip › rubber materials1/data.docx]

Table 1: Summary of Collected 145 CS-RCFST columns database.

| **Ref.** | ***#*** | ***D* (*mm*)** | ***t* (*mm*)** | ***L* (*mm*)** | ***f_y_* (*MPa*)** | ***f_c_′* (*MPa*)** | ***R*** | ***D/t*** | ***L/D*** | ***p_si_*** | ***P_u_*** |
| --- | --- | --- | --- | --- | --- | --- | --- | --- | --- | --- | --- |
| [39] | 16 | 219-219 | 3.0-6.0 | 660 | 215 | 18-36 | 0.1-0.3 | 36.5-73 | 3.01 | 1.4-1.82 | 1715-3310 |
| [5] | 19 | 86-89 | 2.0-3.5 | 188 | 330-342 | 17-86 | 0.0-0.1 | 25.4-43 | 2.11-2.19 | 0.84-1.92 | 409-825 |
| [1] | 3 | 200 | 2.73 | 600 | 331 | 47-62 | 0.0-0.3 | 73.26 | 3.0 | 1.12-1.19 | 2331-2748 |
| [2] | 15 | 114-219 | 2.7-4.25 | 300-500 | 284-456 | 20-42 | 0.0-0.15 | 35.6-57 | 2.28-3.29 | 1.05-1.27 | 484-2888 |
| [40] | 12 | 165 | 2.0-4.0 | 330 | 242 | 10-30 | 0.0-0.3 | 41.3-83 | 2.0 | 1.34-1.68 | 736-1435 |
| [41] | 16 | 114 | 2.0 | 456 | 382 | 3.6-18 | 0.0-0.3 | 57.0 | 4.0 | 1.11-1.54 | 340-638 |
| [3] | 12 | 115-165 | 3.0-4.0 | 345-495 | 231-298 | 24-39 | 0.0-0.3 | 28.8-55 | 3.0 | 1.05-1.56 | 759-1618 |
| [4] | 1 | 86.0 | 2.0 | 188 | 342 | 54.3 | 0.2 | 43.0 | 2.19 | 1.18 | 552 |
| [6] | 3 | 165.1 | 3.5 | 400 | 395 | 14-50 | 0.0-0.3 | 47.17 | 2.42 | 1.08-1.15 | 1130-1876 |
| [42] | 2 | 114.3 | 4.5 | 300-455 | 260 | 15.4 | 0.25 | 25.4 | 2.62-3.98 | 1.36-1.49 | 729-799 |
| [43] | 3 | 125 | 2.5 | 500 | 280 | 27-34 | 0.0-0.15 | 50.0 | 4.0 | 1.42-1.46 | 833-960 |
| [44] | 16 | 87.1 | 1.9 | 253-348 | 255 | 4.8-26 | 0.0-0.35 | 45.84 | 2.9-4.0 | 1.15-1.5 | 180-351 |
| [7] | 6 | 150 | 2.75-5.0 | 330 | 226 | 16-23 | 0.0-0.1 | 30-54.6 | 2.2 | 1.72-2.15 | 1136-1666 |
| [45] | 1 | 114 | 4.8 | 300 | 333 | 41.4 | 0.2 | 23.75 | 2.63 | 1.27 | 1146 |
| [46] | 6 | 152 | 2.8 | 300 | 290 | 6.4-54 | 0.0-0.6 | 54.29 | 1.97 | 1.27-1.31 | 618-1688 |
| [8] | 3 | 101.6 | 1.6 | 300 | 220 | 4.3-11.5 | 0.35-0.75 | 63.5 | 2.95 | 1.09-1.13 | 162-222 |
| [47] | 11 | 114 | 2.0 | 456 | 382 | 17-33 | 0.0-0.1 | 57.0 | 4.0 | 1.03-1.08 | 456-633 |

# is the number of experimental tests for each reference

Table *2*: Total data of Circular rubber aggregate CFST stub columns

| D | t | L | Fy | fc | %R | P |
| --- | --- | --- | --- | --- | --- | --- |
| 219 | 3 | 660 | 215 | 33.067 | 10 | 2269 |
| 219 | 6 | 660 | 215 | 31.067 | 10 | 3104.5 |
| 219 | 6 | 660 | 215 | 26.202 | 20 | 2879 |
| 219 | 3 | 660 | 215 | 24.104 | 20 | 1818 |
| 219 | 6 | 660 | 215 | 23.156 | 30 | 2905 |
| 219 | 3 | 660 | 215 | 23.156 | 30 | 1934.5 |
| 219 | 3 | 660 | 215 | 17.926 | 30 | 1715 |
| 219 | 6 | 660 | 215 | 17.926 | 30 | 2667 |
| 219 | 3 | 660 | 215 | 36 | 10 | 2534 |
| 219 | 6 | 660 | 215 | 34.933 | 10 | 3310 |
| 219 | 6 | 660 | 215 | 30.607 | 20 | 2973.5 |
| 219 | 3 | 660 | 215 | 29.822 | 20 | 2135 |
| 219 | 6 | 660 | 215 | 26.677 | 30 | 2784.5 |
| 219 | 3 | 660 | 215 | 26.677 | 30 | 2065 |
| 219 | 3 | 660 | 215 | 22.682 | 30 | 1813 |
| 219 | 6 | 660 | 215 | 22.682 | 30 | 2742 |
| 89 | 3.5 | 188 | 342 | 63 | 10 | 552 |
| 89 | 3.5 | 188 | 342 | 73.3 | 10 | 700 |
| 89 | 3.5 | 188 | 342 | 74.1 | 10 | 644 |
| 86 | 2 | 188 | 342 | 63 | 10 | 625 |
| 86 | 2 | 188 | 342 | 85.5 | 0 | 764 |
| 86 | 2 | 188 | 342 | 80.1 | 5 | 825 |
| 86 | 2 | 188 | 342 | 73.3 | 10 | 805 |
| 89 | 3.5 | 188 | 342 | 51.5 | 10 | 566 |
| 89 | 3.5 | 188 | 342 | 53.5 | 10 | 601 |
| 86 | 2 | 188 | 342 | 54.1 | 10 | 728 |
| 86 | 2 | 188 | 342 | 56 | 10 | 763 |
| 89 | 3.5 | 188 | 335 | 27.5 | 10 | 501 |
| 89 | 3.5 | 188 | 335 | 35.2 | 10 | 663 |
| 89 | 3.5 | 188 | 335 | 38.3 | 10 | 628 |
| 86 | 2 | 188 | 335 | 31.3 | 10 | 594 |
| 86 | 2 | 188 | 335 | 51.1 | 0 | 681 |
| 86 | 2 | 188 | 335 | 41.6 | 5 | 734 |
| 86 | 2 | 188 | 335 | 41.5 | 10 | 759 |
| 86 | 2 | 188 | 330 | 16.7 | 10 | 409 |
| 200 | 2.73 | 600 | 331.3 | 61.9 | 0 | 2747.8 |
| 200 | 2.73 | 600 | 331.3 | 59.1 | 10 | 2604.5 |
| 200 | 2.73 | 600 | 331.3 | 47.2 | 30 | 2331 |
| 114 | 2.7 | 300 | 284 | 41.60326 | 0 | 723.1 |
| 114 | 2.7 | 300 | 284 | 32.24279 | 5 | 597 |
| 114 | 2.7 | 300 | 284 | 19.70209 | 15 | 483.7 |
| 114 | 3.2 | 300 | 367.7 | 41.60326 | 0 | 930.7 |
| 114 | 3.2 | 300 | 367.7 | 32.24279 | 5 | 826.3 |
| 114 | 3.2 | 300 | 367.7 | 19.70209 | 15 | 673.6 |
| 152 | 2.85 | 500 | 367.7 | 41.60326 | 0 | 1313.7 |
| 152 | 2.85 | 500 | 367.7 | 32.24279 | 5 | 1107.8 |
| 152 | 2.85 | 500 | 367.7 | 19.70209 | 15 | 939.7 |
| 219 | 3.85 | 500 | 284 | 41.60326 | 0 | 2323.5 |
| 219 | 3.85 | 500 | 284 | 32.24279 | 5 | 2197.4 |
| 219 | 3.85 | 500 | 284 | 19.70209 | 15 | 1814.7 |
| 219 | 4.25 | 500 | 455.5 | 41.60326 | 0 | 2887.7 |
| 219 | 4.25 | 500 | 455.5 | 32.24279 | 5 | 2603.6 |
| 219 | 4.25 | 500 | 455.5 | 19.70209 | 15 | 2244.3 |
| 165 | 2 | 330 | 242 | 30.2071 | 0 | 1265.1 |
| 165 | 3 | 330 | 242 | 30.2071 | 0 | 1364.7 |
| 165 | 4 | 330 | 242 | 30.2071 | 0 | 1434.7 |
| 165 | 2 | 330 | 242 | 21.25369 | 10 | 938.6 |
| 165 | 3 | 330 | 242 | 21.25369 | 10 | 1150 |
| 165 | 4 | 330 | 242 | 21.25369 | 10 | 1276.4 |
| 165 | 2 | 330 | 242 | 12.4764 | 20 | 831.2 |
| 165 | 3 | 330 | 242 | 12.4764 | 20 | 1035.5 |
| 165 | 4 | 330 | 242 | 12.4764 | 20 | 1121.8 |
| 165 | 2 | 330 | 242 | 10.05111 | 30 | 736.2 |
| 165 | 3 | 330 | 242 | 10.05111 | 30 | 843.6 |
| 165 | 4 | 330 | 242 | 10.05111 | 30 | 1021.8 |
| 114 | 2 | 456 | 382 | 18.14 | 0 | 638 |
| 114 | 2 | 456 | 382 | 14.47 | 2.5 | 625 |
| 114 | 2 | 456 | 382 | 13.97 | 5 | 583 |
| 114 | 2 | 456 | 382 | 10.64 | 10 | 525 |
| 114 | 2 | 456 | 382 | 7.25 | 20 | 449 |
| 114 | 2 | 456 | 382 | 5.3 | 30 | 404 |
| 114 | 2 | 456 | 382 | 11.05 | 2.5 | 520 |
| 114 | 2 | 456 | 382 | 9.17 | 5 | 516 |
| 114 | 2 | 456 | 382 | 8.45 | 10 | 479 |
| 114 | 2 | 456 | 382 | 5 | 20 | 431 |
| 114 | 2 | 456 | 382 | 3.57 | 30 | 361 |
| 114 | 2 | 456 | 382 | 10.72 | 2.5 | 480 |
| 114 | 2 | 456 | 382 | 8.85 | 5 | 472 |
| 114 | 2 | 456 | 382 | 6 | 10 | 447 |
| 114 | 2 | 456 | 382 | 5.38 | 20 | 368 |
| 114 | 2 | 456 | 382 | 4.09 | 30 | 340 |
| 165 | 3 | 495 | 231 | 38.55 | 0 | 1432.6 |
| 165 | 3 | 495 | 231 | 33.88 | 10 | 1326.4 |
| 165 | 3 | 495 | 231 | 26.48 | 20 | 1372.8 |
| 165 | 3 | 495 | 231 | 23.73 | 30 | 1187.8 |
| 165 | 4 | 495 | 258.13 | 38.55 | 0 | 1617.8 |
| 165 | 4 | 495 | 258.13 | 33.88 | 10 | 1373.6 |
| 165 | 4 | 495 | 258.13 | 26.48 | 20 | 1431.8 |
| 165 | 4 | 495 | 258.13 | 23.73 | 30 | 1385.6 |
| 115 | 4 | 345 | 298.07 | 38.55 | 0 | 827.4 |
| 115 | 4 | 345 | 298.07 | 33.88 | 10 | 759.1 |
| 115 | 4 | 345 | 298.07 | 26.48 | 20 | 770.4 |
| 115 | 4 | 345 | 298.07 | 23.73 | 30 | 776.9 |
| 86 | 2 | 188 | 342 | 54.27 | 20 | 551.8 |
| 165.1 | 3.5 | 400 | 395 | 50.27 | 0 | 1876 |
| 165.1 | 3.5 | 400 | 395 | 24.95 | 15 | 1291 |
| 165.1 | 3.5 | 400 | 395 | 14.37 | 30 | 1130 |
| 114.3 | 4.5 | 300 | 260 | 15.36244 | 25 | 798.65 |
| 114.3 | 4.5 | 455 | 260 | 15.36244 | 25 | 728.95 |
| 125 | 2.5 | 500 | 280 | 34.42652 | 0 | 960 |
| 125 | 2.5 | 500 | 280 | 31.06521 | 5 | 884 |
| 125 | 2.5 | 500 | 280 | 26.84105 | 15 | 833 |
| 87.1 | 1.9 | 252.59 | 255 | 26 | 0 | 345 |
| 87.1 | 1.9 | 252.59 | 255 | 26 | 0 | 351 |
| 87.1 | 1.9 | 252.59 | 255 | 16.66 | 15 | 300 |
| 87.1 | 1.9 | 252.59 | 255 | 16.66 | 15 | 290 |
| 87.1 | 1.9 | 252.59 | 255 | 9.9 | 25 | 250 |
| 87.1 | 1.9 | 252.59 | 255 | 9.9 | 25 | 275 |
| 87.1 | 1.9 | 252.59 | 255 | 4.83 | 35 | 210 |
| 87.1 | 1.9 | 252.59 | 255 | 4.83 | 35 | 200 |
| 87.1 | 1.9 | 348.4 | 255 | 26 | 0 | 325 |
| 87.1 | 1.9 | 348.4 | 255 | 26 | 0 | 340 |
| 87.1 | 1.9 | 348.4 | 255 | 16.66 | 15 | 275 |
| 87.1 | 1.9 | 348.4 | 255 | 16.66 | 15 | 290 |
| 87.1 | 1.9 | 348.4 | 255 | 9.9 | 25 | 220 |
| 87.1 | 1.9 | 348.4 | 255 | 9.9 | 25 | 250 |
| 87.1 | 1.9 | 348.4 | 255 | 4.83 | 35 | 180 |
| 87.1 | 1.9 | 348.4 | 255 | 4.83 | 35 | 190 |
| 150 | 2.75 | 330 | 226 | 23.4 | 0 | 1173 |
| 150 | 2.75 | 330 | 226 | 16.2 | 10 | 1136 |
| 150 | 4 | 330 | 226 | 23.4 | 0 | 1350 |
| 150 | 4 | 330 | 226 | 16.2 | 10 | 1261 |
| 150 | 5 | 330 | 226 | 23.4 | 0 | 1666 |
| 150 | 5 | 330 | 226 | 16.2 | 10 | 1642 |
| 114 | 4.8 | 300 | 333 | 41.4 | 20 | 1146 |
| 152 | 2.8 | 300 | 290 | 54.1 | 0 | 1688 |
| 152 | 2.8 | 300 | 290 | 54.1 | 0 | 1637 |
| 152 | 2.8 | 300 | 290 | 21.2 | 30 | 941 |
| 152 | 2.8 | 300 | 290 | 21.2 | 30 | 944 |
| 152 | 2.8 | 300 | 290 | 6.4 | 60 | 618 |
| 152 | 2.8 | 300 | 290 | 6.4 | 60 | 628 |
| 101.6 | 1.6 | 300 | 220 | 11.45916 | 35 | 222 |
| 101.6 | 1.6 | 300 | 220 | 6.74817 | 50 | 177 |
| 101.6 | 1.6 | 300 | 220 | 4.329014 | 75 | 162 |
| 114 | 2 | 456 | 382.3 | 28.63196 | 0 | 580 |
| 114 | 2 | 456 | 382.3 | 23.73248 | 5 | 527 |
| 114 | 2 | 456 | 382.3 | 21.35867 | 10 | 496 |
| 114 | 2 | 456 | 382.3 | 19.09486 | 10 | 480 |
| 114 | 2 | 456 | 382.3 | 23.20306 | 10 | 522 |
| 114 | 2 | 456 | 382.3 | 17.19798 | 10 | 460 |
| 114 | 2 | 456 | 382.3 | 33.42411 | 10 | 633 |
| 114 | 2 | 456 | 382.3 | 25.94947 | 0 | 530 |
| 114 | 2 | 456 | 382.3 | 17.88566 | 10 | 456 |
| 114 | 2 | 456 | 382.3 | 22.32308 | 10 | 494 |
| 114 | 2 | 456 | 382.3 | 21.79656 | 10 | 502 |
