# Supplementary figures and images for: Circular rubber aggregate CFST stub columns under axial compression: prediction and reliability analysis
Source: Sci Rep. 2024 Oct 31;14:26245. doi: 10.1038/s41598-024-74990-5 (PMC11527877; doi:10.1038/s41598-024-74990-5)

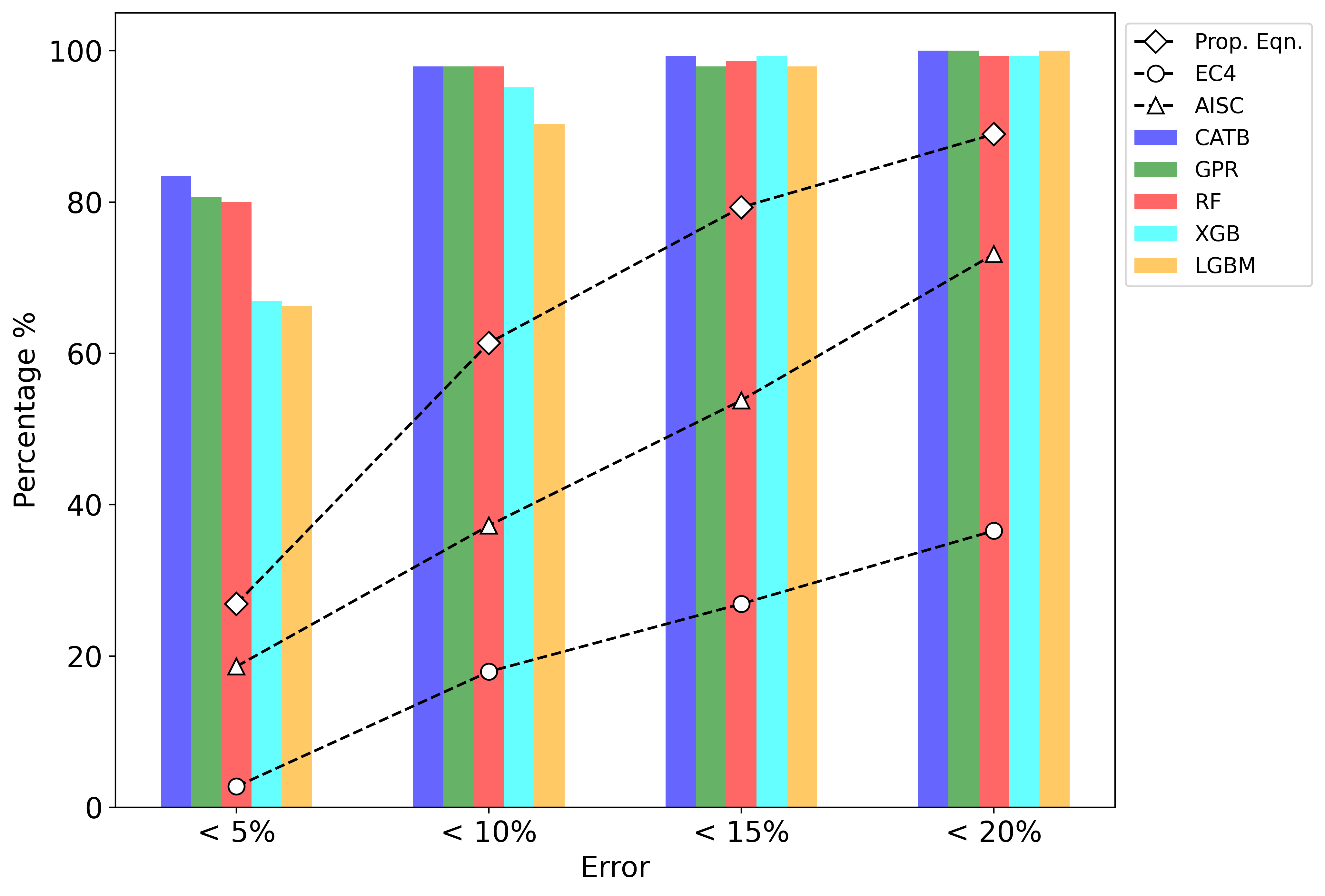

Supplement: Supplementary file 1 — Supplementary Material 1 [file 41598_2024_74990_MOESM1_ESM.zip › rubber materials1/figures/circ.png]

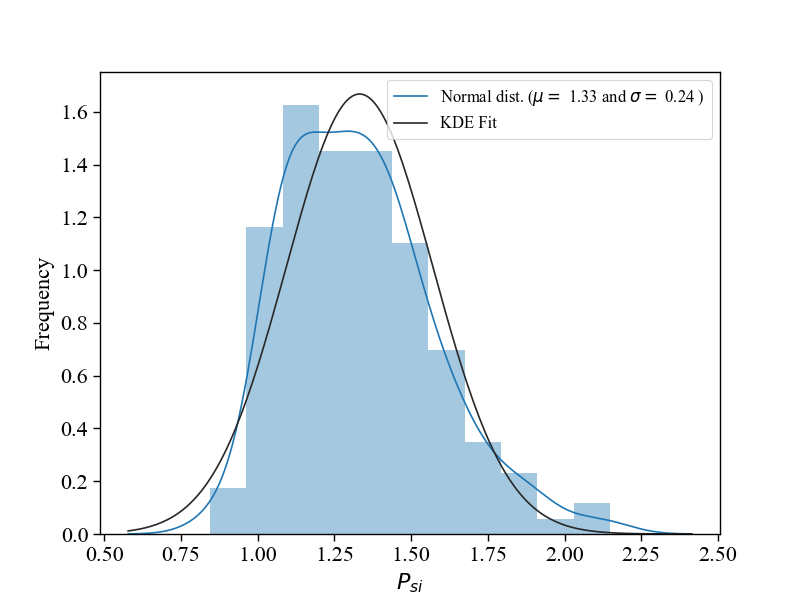

Supplement: Supplementary file 1 — Supplementary Material 1 [file 41598_2024_74990_MOESM1_ESM.zip › rubber materials1/figures/Distribution_circ.png]

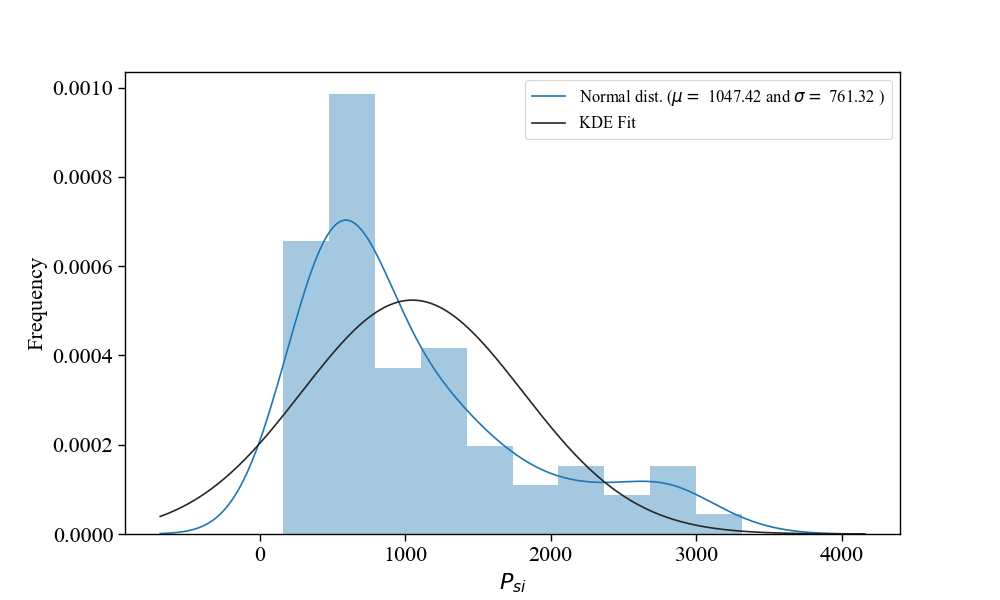

Supplement: Supplementary file 1 — Supplementary Material 1 [file 41598_2024_74990_MOESM1_ESM.zip › rubber materials1/figures/Distribution_circ_before.png]

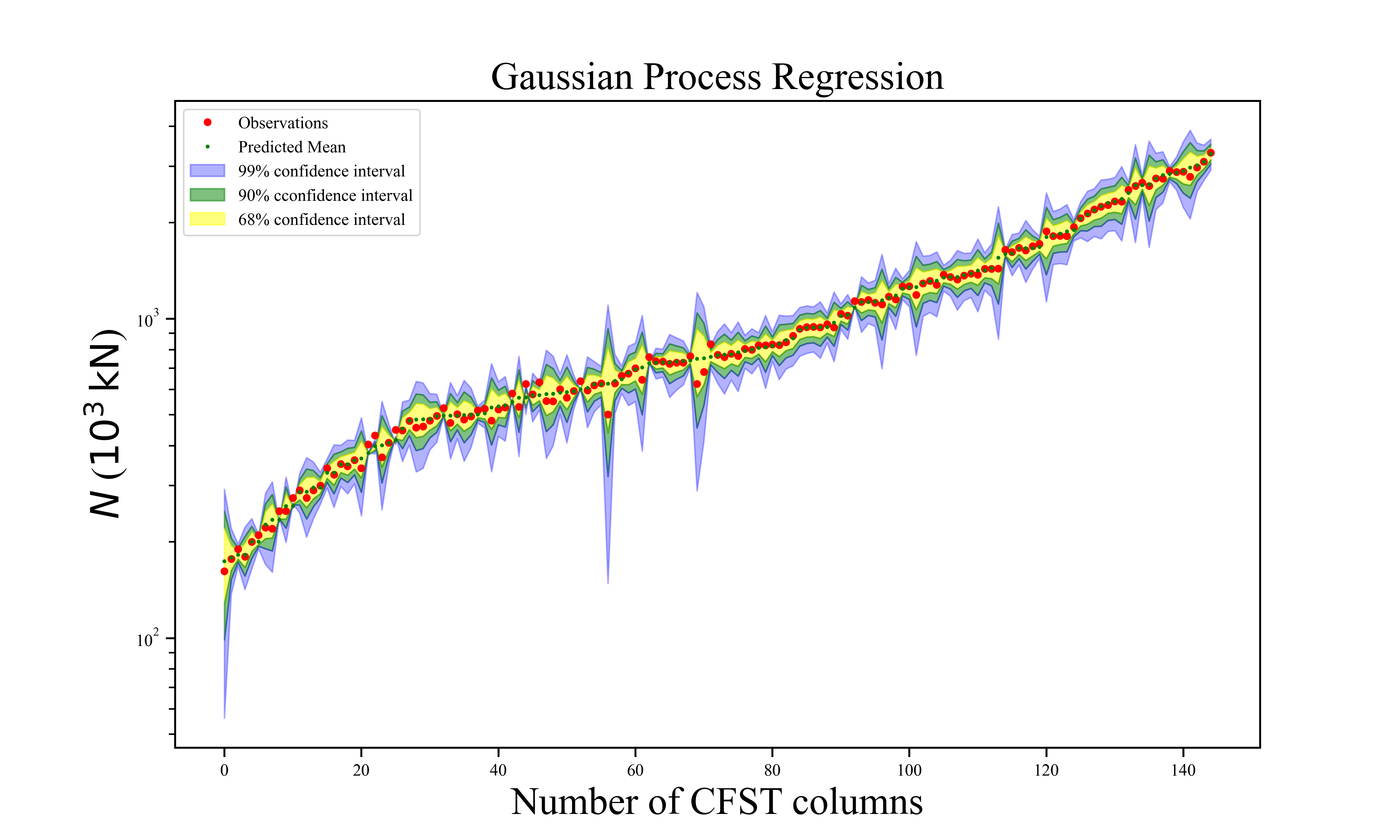

Supplement: Supplementary file 1 — Supplementary Material 1 [file 41598_2024_74990_MOESM1_ESM.zip › rubber materials1/figures/GPR_Predicted_Mean_Confidence_Intervals1i.png]

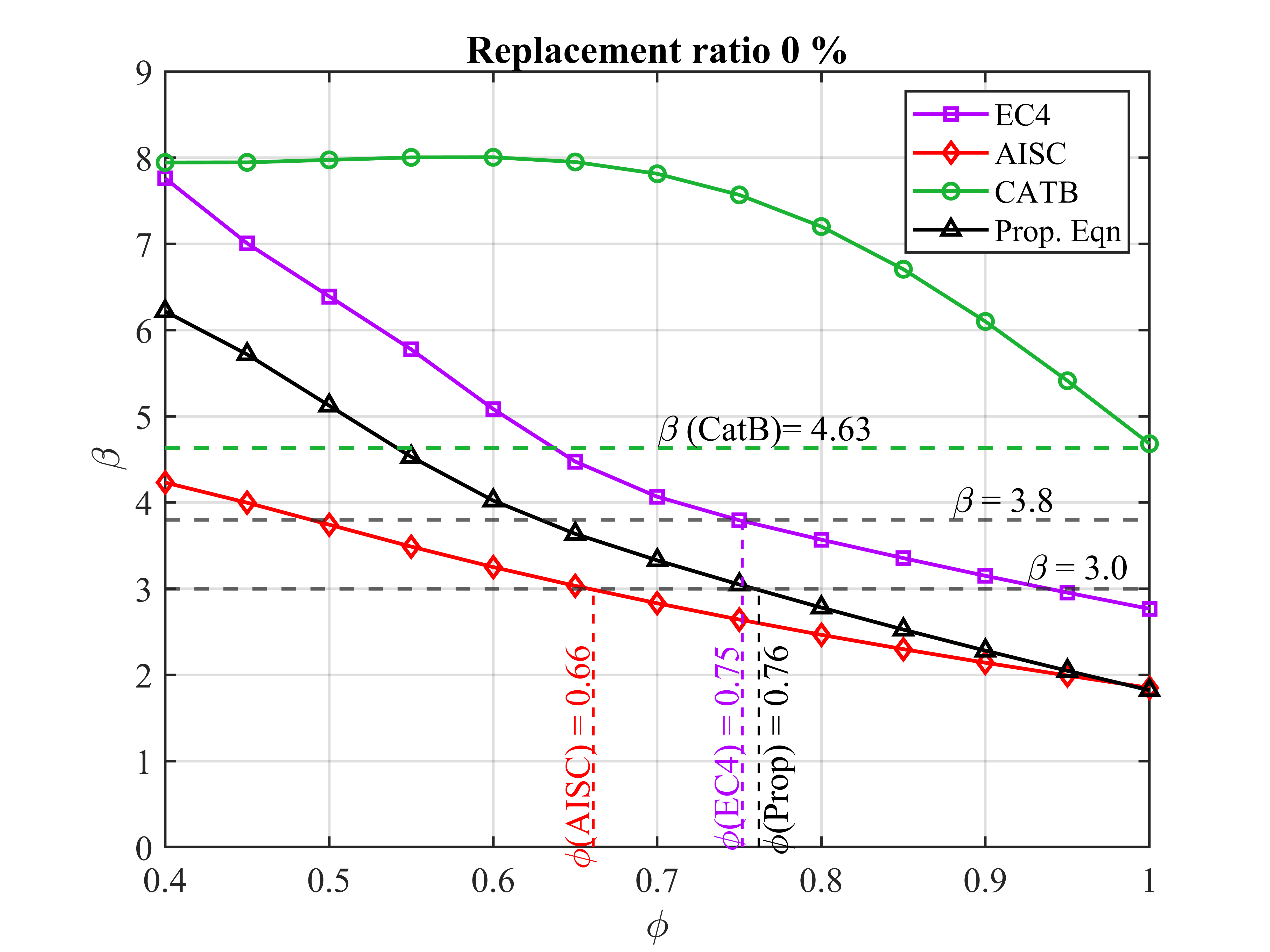

Supplement: Supplementary file 1 — Supplementary Material 1 [file 41598_2024_74990_MOESM1_ESM.zip › rubber materials1/figures/reliabilty1.png]

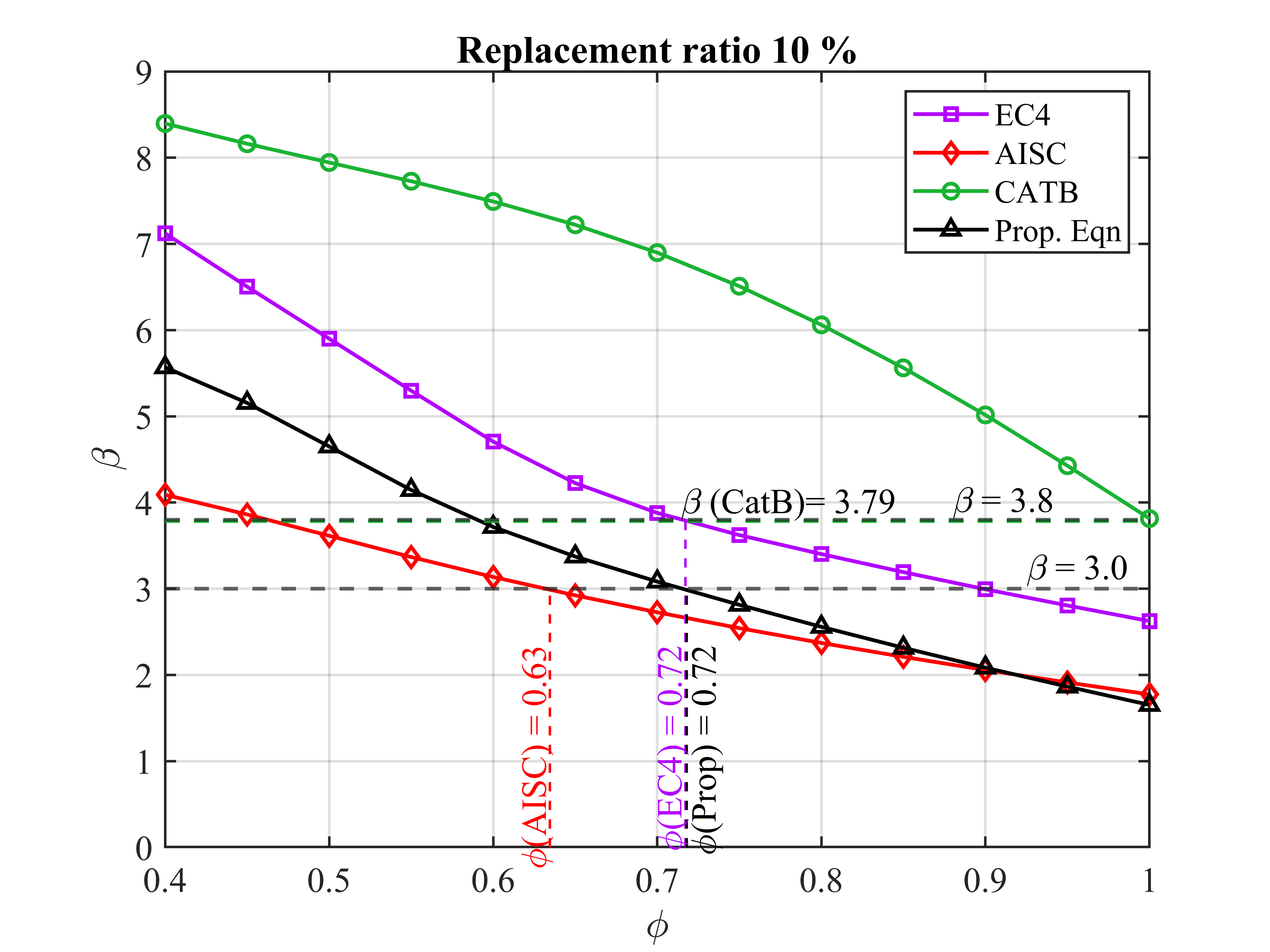

Supplement: Supplementary file 1 — Supplementary Material 1 [file 41598_2024_74990_MOESM1_ESM.zip › rubber materials1/figures/reliabilty2.png]

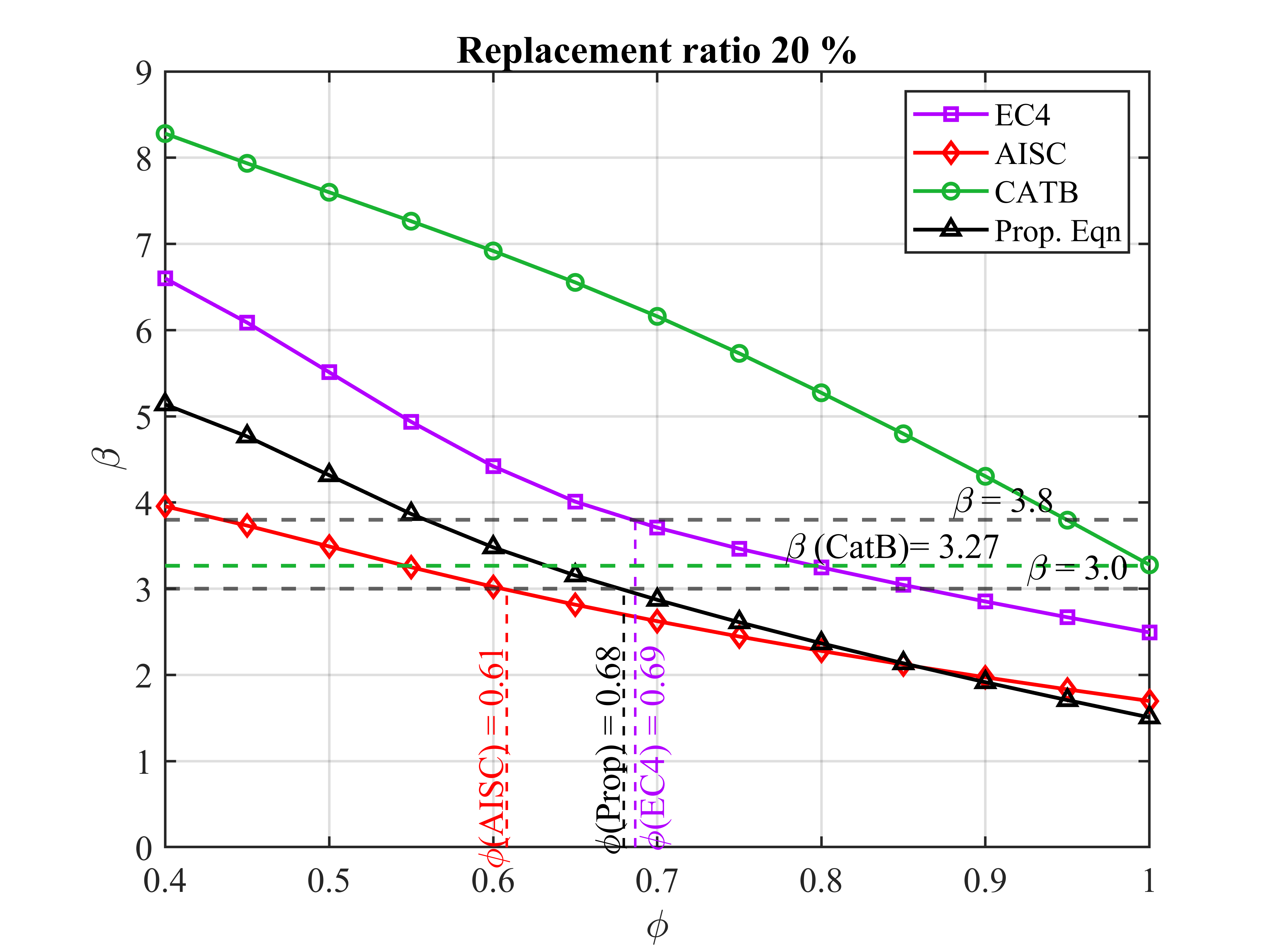

Supplement: Supplementary file 1 — Supplementary Material 1 [file 41598_2024_74990_MOESM1_ESM.zip › rubber materials1/figures/reliabilty3.png]

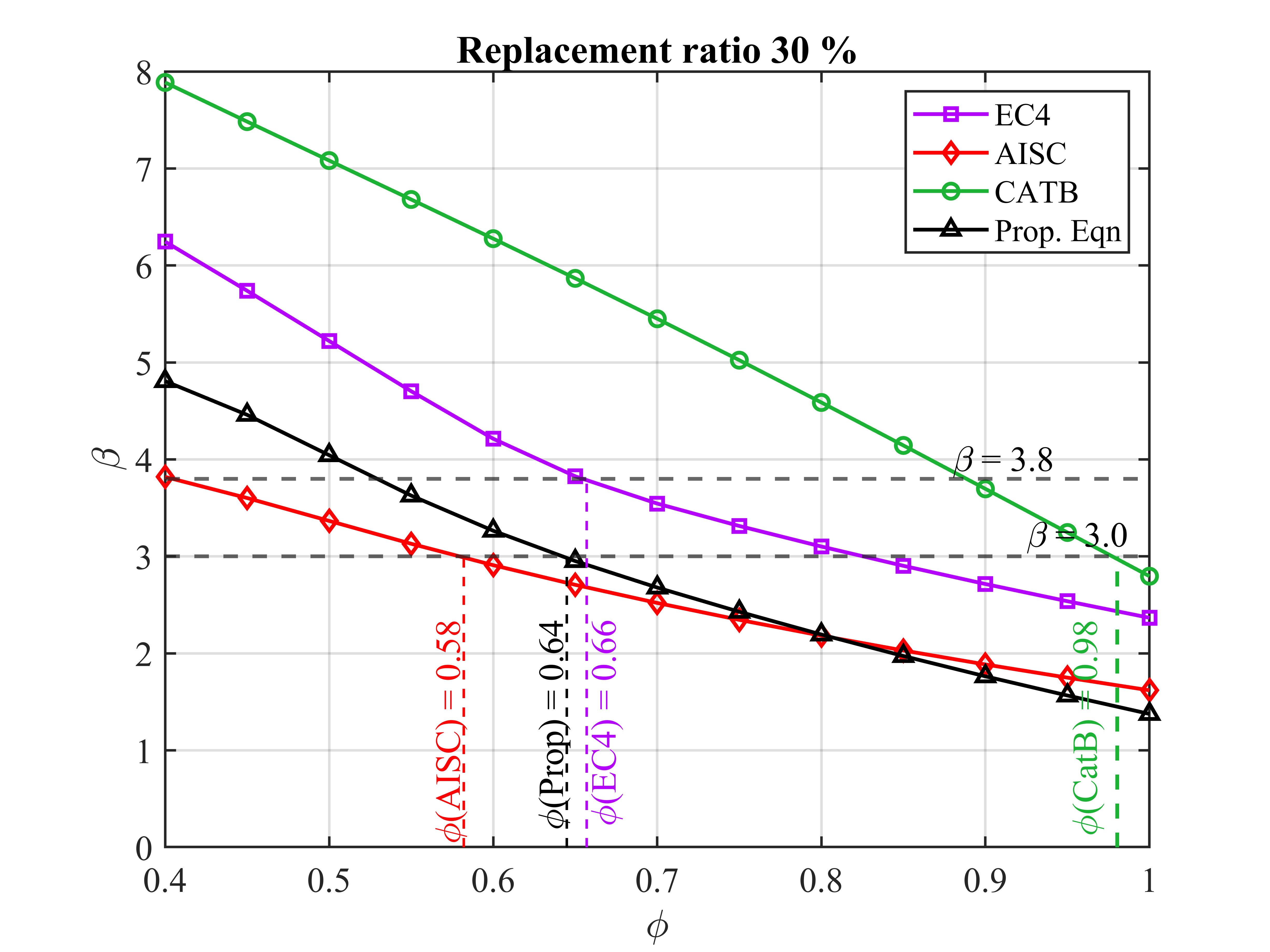

Supplement: Supplementary file 1 — Supplementary Material 1 [file 41598_2024_74990_MOESM1_ESM.zip › rubber materials1/figures/reliabilty4.png]

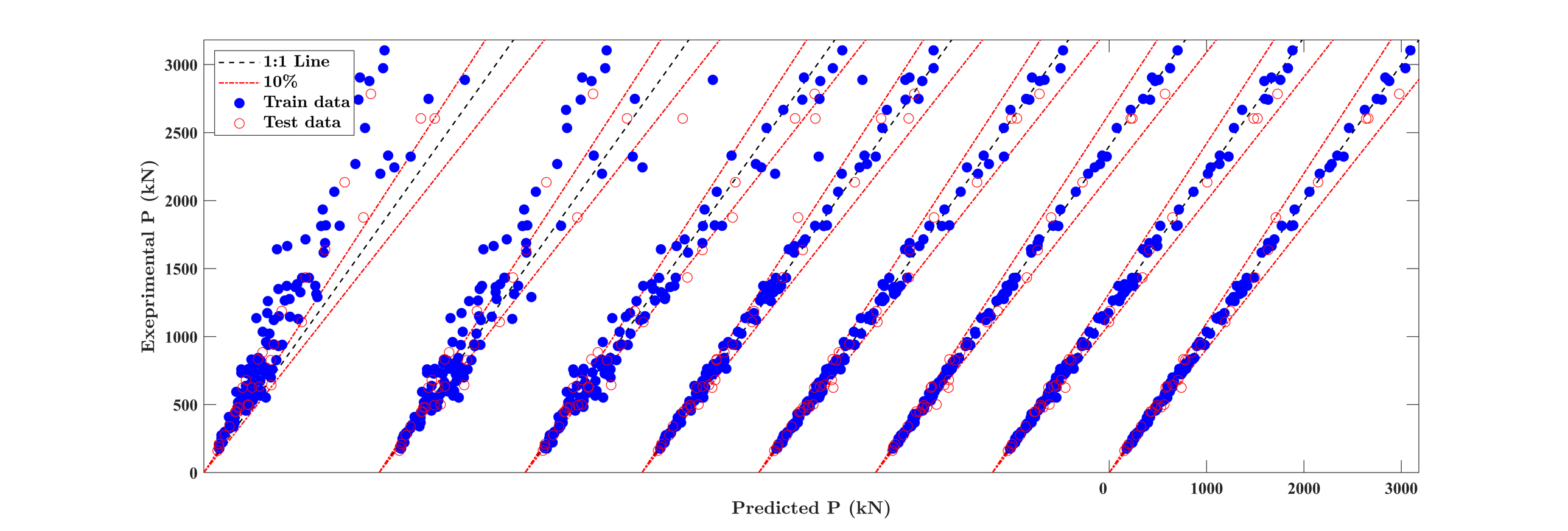

Supplement: Supplementary file 1 — Supplementary Material 1 [file 41598_2024_74990_MOESM1_ESM.zip › rubber materials1/figures/train_test_ML_circc.png]
